# Simulating combined monoaminergic depletions in a PD animal model through a bio-constrained differential equations system

**DOI:** 10.3389/fncom.2024.1386841

**Published:** 2024-08-23

**Authors:** Samuele Carli, Luigi Brugnano, Daniele Caligiore

**Affiliations:** ^1^Computational and Translational Neuroscience Laboratory, Institute of Cognitive Sciences and Technologies, National Research Council (CTNLab-ISTC-CNR), Rome, Italy; ^2^Entersys s.r.l., Padua, Italy; ^3^AI2Life s.r.l., Innovative Start-Up, ISTC-CNR Spin-Off, Rome, Italy; ^4^Department of Mathematics and Computer Science “U. Dini”, University of Florence, Florence, Italy

**Keywords:** Parkinson's disease, treatment, serotonin, noradrenaline, computational model, network neuroscience, locus coeruleus, dorsal raphe nucleus

## Abstract

**Introduction:**

Historically, Parkinson's Disease (PD) research has focused on the dysfunction of dopamine-producing cells in the substantia nigra pars compacta, which is linked to motor regulation in the basal ganglia. Therapies have mainly aimed at restoring dopamine (DA) levels, showing effectiveness but variable outcomes and side effects. Recent evidence indicates that PD complexity implicates disruptions in DA, noradrenaline (NA), and serotonin (5-HT) systems, which may underlie the variations in therapy effects.

**Methods:**

We present a system-level bio-constrained computational model that comprehensively investigates the dynamic interactions between these neurotransmitter systems. The model was designed to replicate experimental data demonstrating the impact of NA and 5-HT depletion in a PD animal model, providing insights into the causal relationships between basal ganglia regions and neuromodulator release areas.

**Results:**

The model successfully replicates experimental data and generates predictions regarding changes in unexplored brain regions, suggesting avenues for further investigation. It highlights the potential efficacy of alternative treatments targeting the locus coeruleus and dorsal raphe nucleus, though these preliminary findings require further validation. Sensitivity analysis identifies critical model parameters, offering insights into key factors influencing brain area activity. A stability analysis underscores the robustness of our mathematical formulation, bolstering the model validity.

**Discussion:**

Our holistic approach emphasizes that PD is a multifactorial disorder and opens promising avenues for early diagnostic tools that harness the intricate interactions among monoaminergic systems. Investigating NA and 5-HT systems alongside the DA system may yield more effective, subtype-specific therapies. The exploration of multisystem dysregulation in PD is poised to revolutionize our understanding and management of this complex neurodegenerative disorder.

## 1 Introduction

Common theoretical and empirical approaches studying Parkinson's disease (PD) focus on dysfunctions in dopamine (DA)-producing cells in the substantia nigra pars compacta. This area projects to the striatum, the principal input gate of the basal ganglia, subcortical nuclei critical to managing motor behavior (Tozzi et al., 2021; Ledonne et al., 2023). Thus, a consistent reduction of striatal DA levels causes malfunctioning of the basal ganglia circuits that, in turn, contribute to the emergence of different PD symptoms (Pare et al., 1990; Dovzhenok and Rubchinsky, 2012; Caligiore et al., 2019). The main PD motor symptoms include resting tremor, bradykinesia, rigidity, and freezing of gait (Jankovic and Kapadia, 2001; Obeso et al., 2010; Caligiore et al., 2019). Cognitive impairments might be evident at diagnosis, even though they significantly manifest in the later stage of the disease progression (Williams-Gray et al., 2007; Aarsland et al., 2009). Moreover, several recent studies suggest that psychiatric disorders, such as depression or anxiety, often develop several years before typical motor symptoms (Faivre et al., 2019). In particular, motivational system dysfunctions manifest early in PD (Pagonabarraga et al., 2015; Cohen et al., 2022; Favier et al., 2022).

Based on this evidence, drug therapies for PD often aim at recovering DA levels (Antonini et al., 2023; Hansen et al., 2023; Pirker et al., 2023). However, while these approaches yield benefits for most PD motor dysfunctions, they generate variable responsiveness for others [e.g., resting tremor (Helmich et al., 2012; Wu and Hallett, 2013; Connolly and Lang, 2014)]. In addition, long-term use of DA may cause adverse effects such as dyskinesia and impulse control disorders (Voon and Fox, 2007; Espay et al., 2018; Castela et al., 2023). The dysfunctional mechanisms leading to PD involve a network of areas and circuits interacting dynamically and influencing each other rather than specific regions and molecular mechanisms working in isolation (Obeso et al., 2010; Caligiore et al., 2016, 2017, 2022; Helmich et al., 2018; Zach et al., 2020). This systemic view is critical for understanding the lack of DA-based therapy consistency. Several studies suggest that, aside from the dopaminergic system, PD could also involve dysfunctions of noradrenergic and serotonergic neuronal populations (Jellinger, 1991; Perez-Lloret and Barrantes, 2016; Wilson et al., 2019; Caligiore et al., 2022; Hezemans et al., 2022). In PD, impairments of locus coeruleus (LC), the dorsal pontine nucleus that synthesizes noradrenaline (NA), begin before nigral pathology and appear to be severe (German et al., 1992; Delaville et al., 2011, 2012). Similarly, the dorsal raphe nucleus (DRN), which is critical for serotonin (5-HT) release, could show impairments earlier than the dopaminergic system and is involved with the development of both non-motor and motor symptoms (Politis and Niccolini, 2015; Jankovic, 2018; Pasquini et al., 2018; Caligiore et al., 2021; Prange et al., 2022).

Starting from this system-level perspective, we propose a bio-constrained computational model that, for the first time, explicitly investigates the neural mechanisms underlying interactions between DA, NA, and 5-HT in a PD animal model. Data on basal ganglia and monoamine areas physiology (Kang and Kitai, 1993; Szabo and Blier, 2001; Liu et al., 2002; Damodaran et al., 2014), including evidence on the effects of NA and 5-HT depletions in a PD animal model (Delaville et al., 2012), constrain key model parameters, hinting at a potential causal dynamical interaction between basal ganglia regions and the areas responsible for neuromodulators release. The model produces predictions on expected activity changes in other brain areas which are not investigated in the target experiments of Delaville et al. (2012). It also highlights the potential efficacy of alternative treatments targeting LC and DRN, yet these are preliminary findings whose effectiveness needs further validation. A sensitivity analysis of the model parameters allows us to identify the critical features affecting the model activity. In this way, it could be possible to frame the most important features affecting the mechanisms underlying the activity of simulated brain areas. Finally, we made a stability analysis that confirms the soundness of the mathematical formulation used to design the model. This point could be critical to validate the effectiveness of the model (Fornari et al., 2020; Shi et al., 2020). It is a relevant aspect that is not readily apparent and seldom explored within computational neuroscience literature. While the soundness of the mathematical formulation is crucial, it becomes irrelevant if the model lacks robust theoretical foundations. Thus, both aspects, mathematical integrity and theoretical underpinnings, serve as indispensable pillars for developing a computational model.

The system-level computational model proposed in this article emphasizes that PD is a multifactorial disorder and opens promising avenues for early diagnosis and subtype-specific treatment tools, harnessing the intricate interactions among monoaminergic systems (Marras et al., 2020; Caligiore et al., 2021; Severson et al., 2021). The investigation into multisystem dysregulation in PD is poised to profoundly transform our comprehension and handling of this intricate neurodegenerative condition.

## 2 Methods

### 2.1 Dynamical model

**Figure 1:**
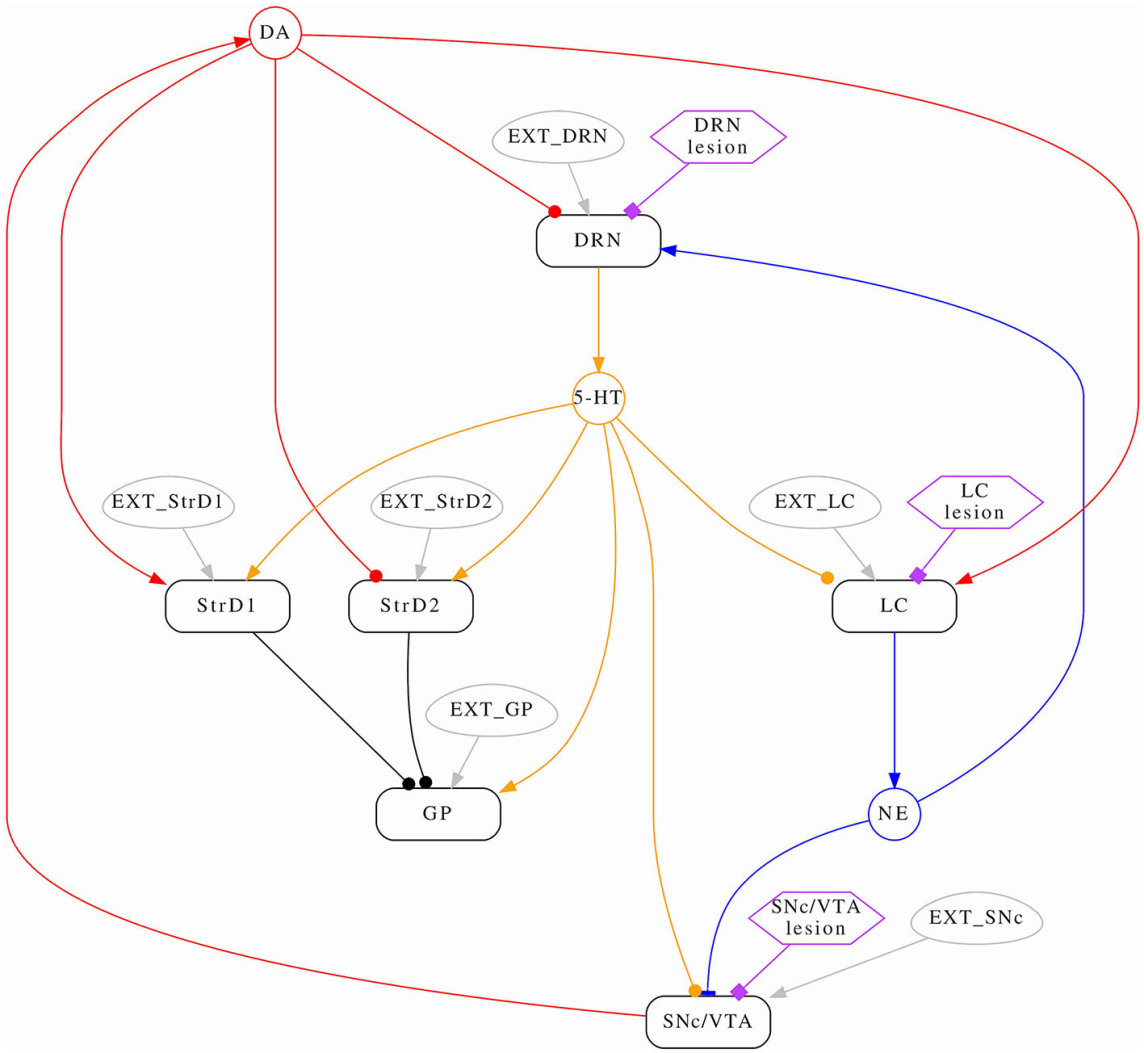
Conceptual model schema. The average activation frequencies of six brain areas are modeled (rounded rectangles); some interactions are modulated by monoamines (circles). Arrows represent positive (excitatory) effects, while circles represent negative (inhibitory) effects. Noradrenaline has a non-linear (both excitatory and inhibitory) effect on SNcVTA which is indicated by a bar. Each area has a corresponding stimulus (ovals) which represents self-activation as well all any other stimulus the area might receive from the rest of the brain which is not modeled. Lastly, hexagons serve as indicators for identifying the areas influenced by the administration of specific drugs.

We introduce a novel model designed to explore the dynamic interactions of three prominent brain monoamines: dopamine (DA), noradrenaline (NA), and serotonin (5-HT), within both typical and pathological contexts. Figure 1 provides an overview of the neural circuitry we assessed. We mimic monoamine efflux by simulating the activation of critical brain regions responsible for initiating such release. Regarding 5-HT/DRN, it is reported that DRN sends projection to striatum (Vertes, 1991; Miyanishi et al., 2023), and we model these projection as excitatory on both direct and indirect pathway. DRN to GP is modeled as excitatory due to serotonin increasing the firing rate of GP neurons as reported in the literature (Chen et al., 2008; Rav-Acha et al., 2008). The projections from DRN to SNcVTA were reported by Gervais and Rouillard (2000) to be dense, and the authors underline the possibility of this connection to be mainly inhibitory; there is a possibility for this projection to be both excitatory and inhibitory but future studies are required to investigate this matter further. Here, we assume this connection to be inhibitory as previously reported by several studies (Dray et al., 1976, 1978; Kelland et al., 1990, 1993). The projection from DRN to LC is also inhibitory (Segal, 1979). StrD1/D2 has inhibitory effects on GP (Nicholson and Brotchie, 2002), while SNcVTA has inhibitory effects on strD2 and excitatory effects on strD1 (Reed et al., 2013); moreover, SNcVTA has excitatory effects on LC as suggested in the literature (Deutch et al., 1986; Lin et al., 2008). The connection from SNcVTA to DRN is modeled as inhibitory because it is reported that after DA depletion, there is an increase of spontaneous activity in DRN (Wang et al., 2009).

The schema from Figure 1 implies the following system of equations:


(1) $$\begin{array}{ll} \dot{\text{GP}} & =-\frac{1}{\tau_{\text{GP}}}\text{GP}-\alpha_{\text{GP}}^{\text{StrD}1}\text{StrD}1-\alpha_{\text{GP}}^{\text{StrD}2}\text{StrD}2+\alpha_{\text{GP}}^{\text{DRN}}\text{DRN}\\ +\alpha_{\text{GP}}^{\text{ext}} \end{array}$$

(2) $$\begin{array}{ll} \dot{\text{StrD}1} & =-\frac{1}{\tau_{\text{StrD}1}}\text{StrD}1+\alpha_{\text{StrD}1}^{\text{SNcVTA}}\text{SNcVTA}+\alpha_{\text{StrD}1}^{\text{DRN}}\text{DRN}\\ +\alpha_{\text{StrD}1}^{\text{ext}} \end{array}$$

(3) $$\begin{array}{ll} \dot{\text{StrD}2} & =-\frac{1}{\tau_{\text{StrD}2}}\text{StrD}2-\alpha_{\text{StrD}2}^{\text{SNcVTA}}\text{SNcVTA}+\alpha_{\text{StrD}2}^{\text{DRN}}\text{DRN}\\ +\alpha_{\text{StrD}2}^{\text{ext}} \end{array}$$

(4) $$\begin{array}{l} \dot{\text{SNcVTA}}=-\frac{1}{\tau_{\text{SNcVTA}}}\text{SNcVTA}-\alpha_{\text{SNcVTA}}^{\text{DRN}}\text{DRN}-\alpha_{\text{SNcVTA}}^{\text{LC}}\text{LC}\\ \;+\beta_{\text{SNcVTA}}^{\text{LC}}{\text{LC}}^{2}\\ +\alpha_{\text{SNcVTA}}^{\text{ext}}\% \end{array}$$

(5) $$\begin{array}{ll} \dot{\text{DRN}} & =-\frac{1}{\tau_{\text{DRN}}}\text{DRN}-\alpha_{\text{DRN}}^{\text{SNcVTA}}\text{SNcVTA}+\alpha_{\text{DRN}}^{\text{LC}}\text{LC}\\ +\alpha_{\text{DRN}}^{\text{ext}} \end{array}$$

(6) $$\begin{array}{ll} \dot{\text{LC}} & =-\frac{1}{\tau_{\text{LC}}}\text{LC}+\alpha_{\text{LC}}^{\text{SNcVTA}}\text{SNcVTA}-\alpha_{\text{LC}}^{\text{DRN}}\text{DRN}\\ +\alpha_{\text{LC}}^{\text{ext}} \end{array}$$

where the abbreviated notation ẋ stands for $\frac{dx}{dt}$ and:

- the time constants τ_*x*_ are all positive and refer to a dampening term which brings back the activity of each area to its resting activation level in the absence of external stimulation (see Table 1);
- the parameters α represent the linear components of the system, are all positive, and follow the notation: $\alpha_{to}^{from}$; $\alpha_{x}^{\text{ext}}$ are synthetic terms that implicitly account for the rest activation of each area and other external stimuli which are not part of the modeled circuit; β is also positive and follows the same notation $\beta_{to}^{from}$ but accounts for non-linear effects;
- the ratios of monoaminic projections from an area to its targets are assumed to be constant.

**Table 1:** Average activity target of the simulated healthy subjects, and time constants used in simulated healthy subjects.

**Area**	**Value**	**Details**
GP	22.0Hz	Globus pallidus (int. and ext. avg.) (Kita and Kita, 2011; Delaville et al., 2012; Liu et al., 2021)
StrD1	10.0Hz	Striatum, Medium spiny neurons type *D*_1_ (Damodaran et al., 2014)
StrD2	9.0Hz	Striatum, Medium spiny neurons type *D*_2_ (Damodaran et al., 2014)
SNcVTA	4.47Hz	Substantia nigra pars compacta and ventro-tegmental area complex (Caligiore et al., 2021)
DRN	1.41Hz	Dorsal raphe nucleus (Caligiore et al., 2021)
LC	2.3Hz	Locus coeruleus (Szabo and Blier, 2001)
**Parameter**	**Value**	**Details**
τ_DRN_	3.3 ± 0.3 ms	Liu et al., 2002
τ_SNcVTA_	1.5 ± 0.3 ms	Kang and Kitai, 1993
τ_LC_	0.8 ± 0.3 ms	Zhang et al., 2010
τ_GP_	18 ± 0.3 ms	Deister et al., 2009
τ_StrD1_	2 ± 0.3 ms	Damodaran et al., 2014
τ_StrD2_	2 ± 0.3 ms	Damodaran et al., 2014

The arbitrary tolerance of ±0.3 has been added to account for the variability observed in the data found in the literature.

### 2.2 Formalization

Let **y** be the status vector of the system of Equations 1–6; we also define *s* to be the size of **y**, hence the number of equations in the system. We therefore have


(7) $$\begin{array}{l} \text{y}={\left(\text{GP},\text{StrD}1,\text{StrD}2,\text{SNcVTA},\text{DRN},\text{LC}\right)}^{T}\in {\mathbb{R}}^{s} \end{array}$$

The system is autonomous and can therefore be represented in the form:


(8) $$\begin{array}{l} \dot{\text{y}}\left(t\right)=\text{f}\left(\text{y}\left(t\right)\right) \end{array}$$

where each component of the function **f**:(ℝ × ℝ^*s*^) → ℝ^*s*^ is defined by the corresponding equation in Equations 1–6, and where we assume the initial state **y**(*t*_0_) = **y**_0_ to be known.

Starting from Equation 8, Equations 1–6, 8 can be represented in matrix form:


(9) $$\begin{array}{l} \dot{\text{y}}\left(t\right)=A\text{y}\left(t\right)+C\left(\text{y}\left(t\right).\text{y}\left(t\right)\right)+\text{b}, \end{array}$$

where *a*_*ij*_ = 0 if the corresponding $\alpha_{i}^{j}$ is not defined and likewise *b*_*i*_ = 0 if $\alpha_{i}^{ext}$ is not defined, and also $c_{ij}=\beta_{i}^{j}$ with *c*_*ij*_ = 0 if the corresponding $\beta_{i}^{j}$ coefficient is not defined; “°” indicates the element-wise vector product (or Hadamard).

### 2.3 Modeling lesions

Each element within the status vector (Equation 7) corresponds to the average activation frequency of the corresponding brain region. This, in turn, serves as an indirect indicator of the production and projection of monoamines to the affected areas. We posit that a depletion in monoamines results from the death or temporary incapacitation of a portion of neurons within a given area, which is directly manifested as a decrease in the average activation frequency of that area. Consequently, when the SNcVTA is lesioned, we observe a reduction in DA levels; lesioning the DRN results in a decrease in 5-HT, and lesioning the LC leads to a reduction in NA levels.

Each equation of the model is composed by three conceptual blocks: a damping term, a constant stimulus, and a reaction to projections from other areas. The constant stimulus represents external and internal activation sources that are not directly accounted for in this model. Together with the damping term, the constant stimulus accounts for the resting behavior of the area: The area will stabilize to its rest activation frequency. In absence of reaction terms, each equation has an equilibrium point:


(10) $$\begin{array}{l} y^{\prime }\left(t\right)=-\frac{1}{\tau }y\left(t\right)+k,\;y^{\prime }\left(t\right)\equiv 0\Rightarrow y\left(t\right)=k\tau . \end{array}$$

The time constants τ are derived from the literature (see Table 1), and we assume them to be typical values for the specific kind of neuron found in an area; we therefore assume that they are not altered by the lesion. It is, however, reasonable to expect that the sensitivity of lesioned area to internal and external stimuli will change in such a way that the average activation frequency changes to the levels which have been experimentally measured.

We can now define multiple versions of the same model, which differ from the healthy model only for the constant term and reaction coefficients of the lesioned area. For example, suppose a healthy subject is modeled using model (Equation 9) by the coefficients held in *A, C*, **b**. Having received a dopaminergic lesion (hence, SNcVTA neurons are malfunctioning), the subject will now be modeled by the same equations of model (Equation 9) but this time with coefficients *A*_*LDA*_, *C*_*LDA*_, **b**_*LDA*_, which differ by *A, C*, **b** only by the values corresponding to the parameters of the equation for SNcVTA, namely, $\alpha_{\text{SNcVTA}}^{\text{DRN}}$, $\alpha_{\text{SNcVTA}}^{\text{LC}}$, $\beta_{\text{SNcVTA}}^{\text{LC}}$, $\alpha_{\text{SNcVTA}}^{\text{ext}}$. Likewise, when the subject also receives a serotonergic lesion, there will be a third set of parameters *A*_*LDA*+*L*5*HT*_, *C*_*LDA*+*L*5*HT*_, **b**_*LDA*+*L*5*HT*_ which again differ from *A*_*LDA*_, *C*_*LDA*_, **b**_*LDA*_ only by the parameters corresponding to the equation for DRN, and so on.

A single subject is therefore represented by multiple versions of the parameters matrices *A, C*, **b**, each set corresponding to one particular state: healthy (also called SHAM), LDA, L5HT, LNE when only one of the lesions is applied, LDA + L5HT, LDA + LNE when lesions are combined, and so on.

### 2.4 Stability conditions

The solution trajectories of a dynamical system are referred to as “stable” when small perturbations of the initial conditions lead to trajectories which have fundamentally the same behavior of the unperturbed ones and differ from the latter in a proportional way with respect to the the perturbation magnitude. An unstable system can otherwise have a high sensitivity to such perturbation, which originate wildly varying solution behavior. At the limit of instability, there are chaotic systems, for which very small perturbation of the initial conditions can lead to completely different dynamical behavior. Stability is a property of a particular solution (Kelley and Peterson, 2001; Lakshmikantham and Trigiante, 2002; Riley et al., 2006; Press, 2007; Butcher, 2016).

A system which has stable solution trajectories can have great predictive power since its low sensitivity to the initial conditions results in solutions which have comparable errors. Chaotic systems, on the other hand, are so sensitive to the errors on the initial conditions that it is effectively impossible to use them for obtaining useful predictions. For instance, weather is a chaotic system, and that is why it is so difficult to compute reliable long-term weather predictions. Fortunately enough, the aspects of brain chemistry that we are simulating in this study do not exhibit chaotic behavior; on the contrary, they exhibit self-regulation and great resilience to perturbations. It makes therefore sense to require them to be simulated using a system of equations having asymptotically stable equilibria.

Equation 9 has at least one equilibrium point $\bar{\text{y}}$ such that:


(11) $$\begin{array}{l} \text{0}=A\bar{\text{y}}+C\left(\bar{\text{y}}.\bar{\text{y}}\right)+\text{b} \end{array}$$

The stability properties of the equilibrium point in Equation 11 are analyzed in details in Appendix 1; the resulting stability conditions are employed as one of the components of the fitness measure of the model, as described in Section 2.7. In this way, it is ensured that all the solution trajectories simulated by the model are asymptotically stable. Consequently, the numerical trajectories exhibit the expected behavior even for long-time simulations.

## 3 Simulation setup

### 3.1 Free parameters and constants

As hinted in Sections 2.3 and 3.3, we require a model to be able to reproduce its target data in four different states at the same time: healthy (SHAM), dopaminergic lesion (LDA), noradrenergic lesion (LNE), and serotonergic lesion (L5HT). The combinations LDA + LNE and LDA + L5HT are instead constrained only to a target range, to be able to also serve as a prediction (and hence as a measure of the agreement of the model with experimental data). Below, we briefly outline the mathematical formalism used to assess the model's stability properties and to establish the simulation setup (Equations 12–18).

Let *S*_*i*_ be the set of parameters that define the model representing test subject *i*. *S*_*i*_ contains the following:

- Six time constants: τ_GP_, τ_StrD1_, τ_StrD2_, τ_SNcVTA_, τ_DRN_, τ_LC_. As discussed in Section 2.3, the time constants are derived from the literature and are not optimized;
- SHAM: the healthy model has 20 free parameters, namely, all α and β parameters defined in Equations 1–6;
- LDA: the dopaminergic lesion instance has four free parameters, that is, $\alpha_{\text{SNcVTA}}^{\text{DRN}}$, $\alpha_{\text{SNcVTA}}^{\text{LC}}$, $\beta_{\text{SNcVTA}}^{\text{LC}}$, $\alpha_{\text{SNcVTA}}^{\text{ext}}$. Those are all the parameters of the SNcVTA equation. All the other parameters are kept constant and are the same as in SHAM;
- L5HT: the serotonergic lesion instance has three free parameters, that is, $\alpha_{\text{DRN}}^{\text{SNcVTA}}$, $\alpha_{\text{DRN}}^{\text{LC}}$, $\alpha_{\text{DRN}}^{\text{ext}}$. All the other parameters are kept constant and are the same as in SHAM;
- LNE: the Noradrenergic lesion has three free parameters, that is, $\alpha_{\text{LC}}^{\text{SNcVTA}}$, $\alpha_{\text{LC}}^{\text{DRN}}$, $\alpha_{\text{LC}}^{\text{ext}}$. All the other parameters are kept constant and are the same as in SHAM;
- LDA + L5HT, LDA + LNE: the combination of lesions do not have any free parameters but are constructed by applying to the SHAM values, in order, the relevant values from each lesion.

The set *S*_*i*_ therefore contains a total of 36 parameters, 30 of which must be optimized at the same time to fit the available data. Appropriate subsets of the parameters in *S*_*i*_ are then used to build the corresponding matrices *A, C*, **b** to completely define (Equation 9). and hence compute its solution and properties.

### 3.2 Model fitness definition

We will hereafter refer to *S*_*i*_ as the complete model for subject *i*, since it is the set of parameters that completely define it. The variations $S_{i}^{\text{kind}}$, such as $S_{i}^{\text{SHAM}}$ and $S_{i}^{\text{LDA}}$, will refer instead to the subset of parameters which are currently being applied to actually simulate the model.We will denote one solution as


(12) $$S_{i}^{\text{SHAM}}(y_{0},t_{0},T)=Y=\left(\begin{array}{ccc} y_{1}(t_{0}) & \cdots & y_{1}(t_{N})\\ \vdots & \; & \vdots \\ y_{s}(t_{0}) & \cdots & y_{s}(t_{N}) \end{array}\right)$$

*Y* is therefore the solution obtained by integrating the model in the interval [*t*_0_, *T*], with the starting vector **y**_0_, and using the SHAM subset of parameters. The matrix *Y* comprises s rows, and the *i*-th row corresponds to the *i*-th equation in the system of differential equations.

The number *N* of integration steps, as well as their size, is usually variable and chosen by the integration method case-by-case; hence, it can potentially be different for each subset of parameters.

Likewise, we will denote with $T_{i}^{kind}$ the corresponding reference solutions that will be used to evaluate the fitness of the model:


(13) $$T_{i}^{SHAM}(J)=Y_{T}(J)=\left(\begin{array}{ccc} y_{T1}(t_{0}) & \cdots & y_{T1}(t_{N})\\ \vdots & \; & \vdots \\ y_{Ts}(t_{0}) & \cdots & y_{Ts}(t_{N}) \end{array}\right)$$

where *J* = [*t*_0_, ..., *t*_*n*_] is a vector of times. The fitness of a model is finally obtained as a combination of many fitness figures *f*_*i*_∈[0, 1], which measure a wide range of properties of the simulated solution with respect to the reference ones. In more detail:


(14) $$\begin{array}{l} f=\sqrt{\underset{i}{\min}\left(f_{i}\right)\frac{1}{n}\overset{n}{\underset{i=1}{\sum }}f_{i}},\;f_{i}\in F,\;n=|F|. \end{array}$$

Details about the fitness computations are reported in Appendix 2.

### 3.3 Synthetic reference data

The average brain activation in healthy subjects, as well as the time constants, has been extracted from the literature and are reported in Table 1. Starting from these average values, we craft a synthetic activity profile for a population of subjects (240 simulated subjects). This is achieved by modeling a normal distribution centered around the average value, with a normalized maximum excursion of ±50%.

In the reference (or target) data that we aim to replicate using the computational model, a population of adult male rats is subdivided into six groups:


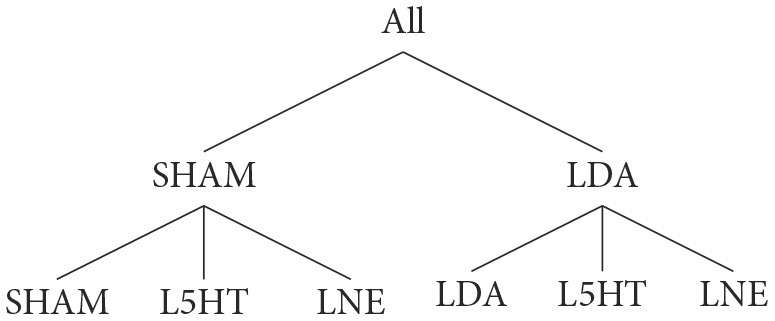


where:

- SHAM: indicates that subjects are treated with saline;
- LDA: dopamine depletion;
- LNE: noradrenaline depletion;
- L5HT: serotonin depletion.

Table 2 summarizes the reference changes in GP activity.

**Table 2:** Reference data for area interaction and lesion effects.

**Group**	**Frequency**	**Interval**
SHAM	22Hz	
SHAM + LDA	22Hz	= SHAM (Kita and Kita, 2011)
SHAM + LNE	22Hz	= SHAM
SHAM + L5HT	15Hz	(= 0.65 SHAM)
LDA + LNE		(0.5 SHAM ≤ x ≤ SHAM)
LDA + L5HT		(0.5 SHAM ≤ x ≤ SHAM)
**Group**		**Lesion Effect**
SHAM+LDA		SNcVTA drops by at least 90% compared to SHAM
		LC drops by at least 20% compared to SHAM (Szabo and Blier, 2001)
SHAM+LNE		LC drops by at least 80% compared to SHAM
SHAM+L5HT		DRN drops by at least 70% compared to SHAM

**Figure 2:**
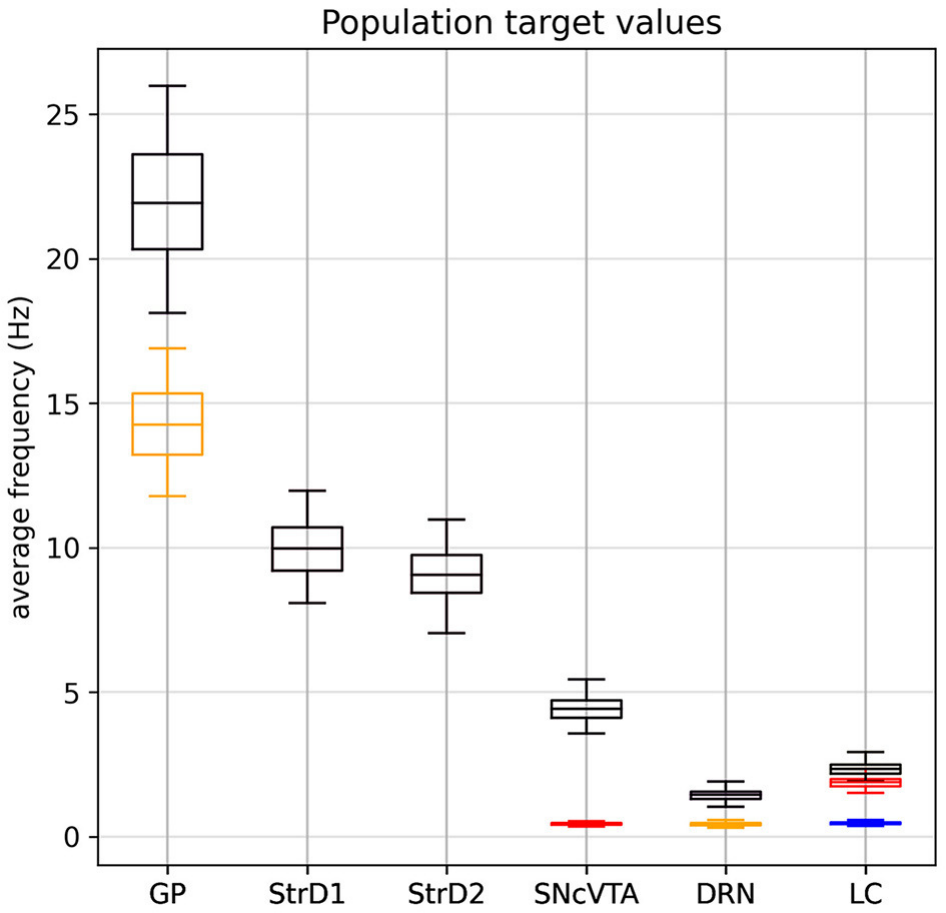
Generated target value distribution for all cases: in black, the SHAM case. The LDA dopaminergic lesion target (red) differs from SHAM only for SNcVTA and LC. The serotonergic L5HT lesion target (yellow) differs from SHAM only in for GP and DRN. Finally, the noradrenergic LNE lesion target (blue) differs from SHAM only for LC. Values for each individual are generated according to Section 3.3: Each area follows a normal distribution around a center value with a maximum spread of ±50% (4σ = 0.5μ). Lesioned activation values of an area, when defined, are scaled according to Table 2.

The experimental data we have collected, summarized in Tables 1, 2, ultimately consist of normal distributions around their respective center values which are to be considered constant; we do not have explicit information about the dynamic behavior of the system or about the transition from a state to the other. Of course, each subject must go through a dynamic transition from a healthy state to a lesioned state, but both states must be asymptotically stable solutions for the model. Since there is no single study that lists all the required brain area activation values for a particular subject at the same time, we have no choice but to generate a synthetic population of virtual subjects with area activation values which lie within the distributions identified across the literature. The generated target value distributions for all cases are summarized in Table 3 and Figure 2.

**Table 3:** Target values.

**Area**	**Value**
GP	$\leftarrow N\left(22,22\cdot \frac{1}{8}\right)$
GP^*LDA*^	= GP
GP^*L*5*HT*^	= GP·0.65
GP^*LNE*^	= GP
GP^*LDA*+*L*5*HT*−max^	= GP·0.75
GP^*LDA*+*L*5*HT*−min^	= GP·0.65
GP^*LDA*+*LNE*−max^	= GP
GP^*LDA*+*LNE*−min^	= GP·0.65
StrD1	$\leftarrow N\left(10,10\cdot \frac{1}{8}\right)$
StrD2	$\leftarrow N\left(9,9\cdot \frac{1}{8}\right)$
SNcVTA	$\leftarrow N\left(4.47,4.47\cdot \frac{1}{8}\right)$
SNcVTA^*LDA*^	= SNcVTA·0.1
DRN	$\leftarrow N\left(1.41,1.41\cdot \frac{1}{8}\right)$
DRN^*L*5*HT*^	= DRN·0.3
LC	$\leftarrow N\left(2.3,2.3\cdot \frac{1}{8}\right)$
LC^*LDA*^	= LC·0.8
LC^*LNE*^	= LC·0.2

In particular, we generate a number of subjects *S*_*i*_ and each of them is associated with a set of target values *T*_*i*_ which are defined as described in Table 3, where x←N(μ,σ) indicates that *x* is a random number drawn from a normal distribution using the provided parameters. Given the synthetic nature of this data, it is reasonable to assume activities of each area to have the same distribution. We impose every value to lie within ±50% of the center value by imposing $4\sigma =\frac{1}{2}\mu$. Since data from literature can be interpreted as an average percentage change in activity for lesioned areas, we decided to treat the generated healthy value for an area as the reference level of an individual and use that as a base to generate the lesioned values that where needed. In this way, a subject that has a higher than average value for an area in healthy conditions will also have an higher than average level in the same area when lesioned, although the value will change by the required proportional amount.

Reference solutions for subject *i* are therefore composed of constant values:


(15) $$T_{i}^{d}(J)=\left(\begin{array}{ccc} GP^{d} & \cdots & GP^{d}\\ \vdots & \; & \vdots \\ LC^{d} & \cdots & LC^{d} \end{array}\right)\in \;{\mathbb{R}}^{s\times n+1}$$

where *d* is one of SHAM, LDA, L5HT, LNE, LDA+L5HT, LDA+LNE; the appropriate value of each area for the respective lesion is chosen according to *d* when available, otherwise defaults to the SHAM (not labeled) value.

### 3.4 Choosing the simulation time

The simulation time is arbitrarily set to 0.5s under the assumption that the basic behavior of each equation in the system will resemble (Equation 10); hence, the transition time between any state to a stable solution will be dominated by the slowest time constant (which is derived from the literature). In fact, since the solution to:


(16) $$\begin{array}{l} y^{\prime }\left(t\right)=-\frac{1}{\tau }y\left(t\right)+k \end{array}$$

assuming *y*(0) = 0, is


(17) $$\begin{array}{l} y\left(t\right)=-k\tau e^{-\frac{t}{\tau }}+k\tau \end{array}$$

we can compute the time it takes for the solution to grow past 99% of its limit value:


(18) $$\begin{array}{l} 0.99k\tau =-k\tau e^{-\frac{t}{\tau }}+k\tau \Rightarrow t=\log\left(0.01\right)\tau \approx 5\tau \end{array}$$

(which in engineering contexts is broadly known as the “rule of the five taus").

In this specific case, the slowest τ ≤ 20ms, therefore we can assume the transient phase to be finished after 5·0.02s = 0.1s, and a time 5 times longer, 0.5s, should be adequate to see a long stable steady state, and we would expect the dynamic behavior to have stabilized already around 0.1s.

### 3.5 Model parameters dimensionality analysis

Each component of the status vector **y** (Equation 9) directly represents the average activation frequency of a brain area and is therefore expressed in Hz. The derivative terms in each equation of Equations 1–6 are all derivatives with respect to time of a frequency; hence, they are all expressed in Hz/s (or 1/s^2^). Consequently, the external stimulus parameters α^ext^ must also be expressed in Hz/s, while the remaining α parameters must be 1/s, hence Hz. The second order term parameter β is instead a pure number, since Hz^2^ =1/second^2^ =Hz/s. Finally, all time constants τ are naturally expressed in seconds.

### 3.6 Simulation and optimization

All simulations are obtained using a variable order, variable step scheme backward-differentiation formulas (BDF) (Byrne and Hindmarsh, 1975) integrator provided by the Python scipy library. The optimization of parameters is then performed using differential evolution (DE), also as provided by the Python scipy package, with a population of 240 simulated subjects, DE/best/1/exp strategy with *C*_*r*_ = *F* = 0.95 initialized with a uniform Halton distribution.

An external optimization cycle which sets different random generator seeds has been used to retry the cases which did not find convergence. In so doing, all cases did eventually converge after a few attempts. The full codebase can be found at https://github.com/WohthaN/Simulating_noradrenaline_and_serotonin_depletions_in_parkinson.

## 4 Results

**Figure 3:**
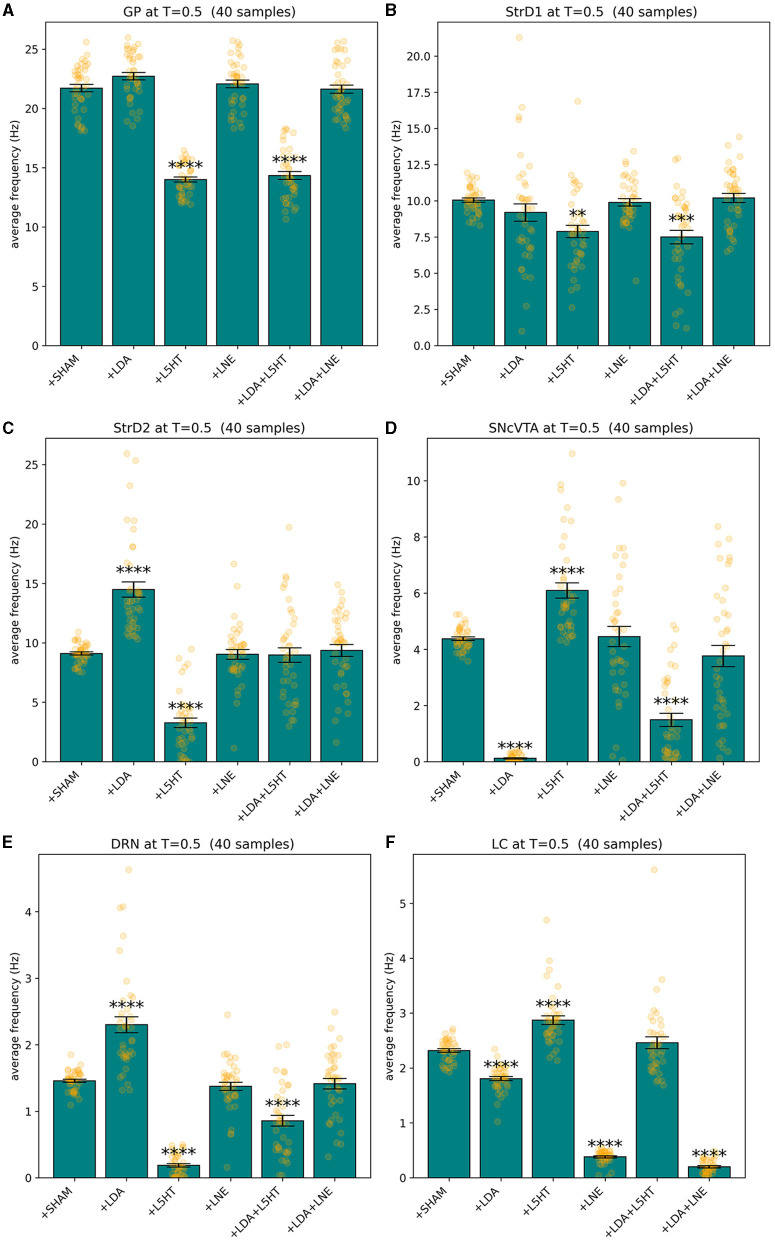
Summary of the behavior exhibited by GP **(A)**, StrD1 **(B)**, StrD2 **(C)**, SNcVTA **(D)**, DRN **(E)**, and LC **(F)** across various groups including SHAM, LDA, L5HT, LNE, LDA + L5HT, and LDA+LNE, with each group consisting of 40 distinct subjects binned accordingly. ^**^p ≤ 0.01, ^***^p ≤ 0.001, and ^****^p ≤ 0.0001.

All models fit the corresponding target values as defined in Table 3 with a fitness *f*≥1 − 10^−8^. Since the measure is dominated by the smallest fitness value being combined by definition, also the mean square difference of each component of the solution from its reference value is bonded by the same order of magnitude, which is a suitable precision for the purposes of this study. Figure 3 shows an overview of the simulated behavior of all six areas in all conditions. The model reproduces the target data with animals presented in Delaville et al. (2012). In all cases, the simulated values for the SHAM case overlap the target values with the imposed tolerance. In the lesion groups, all areas which do not have a target defined are model predictions. All instances of the model meet the stability conditions defined in Section 2.4 as imposed by the fitness measure.

**Figure 4:**
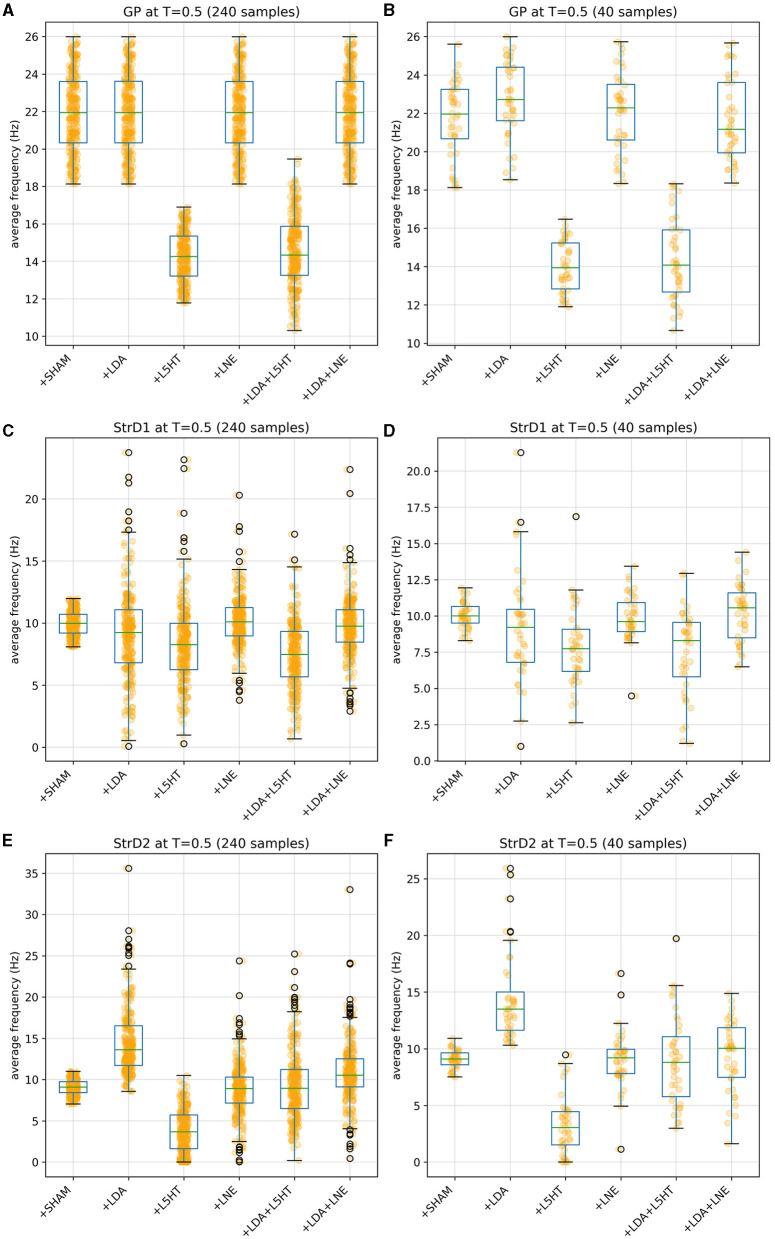
Distribution of simulated equilibrium points for the GP **(A)**, StrD1 **(C)**, and StrD2 **(E)** regions across the entire synthetic population. On the right, the simulated equilibrium points for GP **(B)**, StrD1 **(D)**, and StrD2 **(F)** are depicted within separate bins, each containing 40 samples.

**Figure 5:**
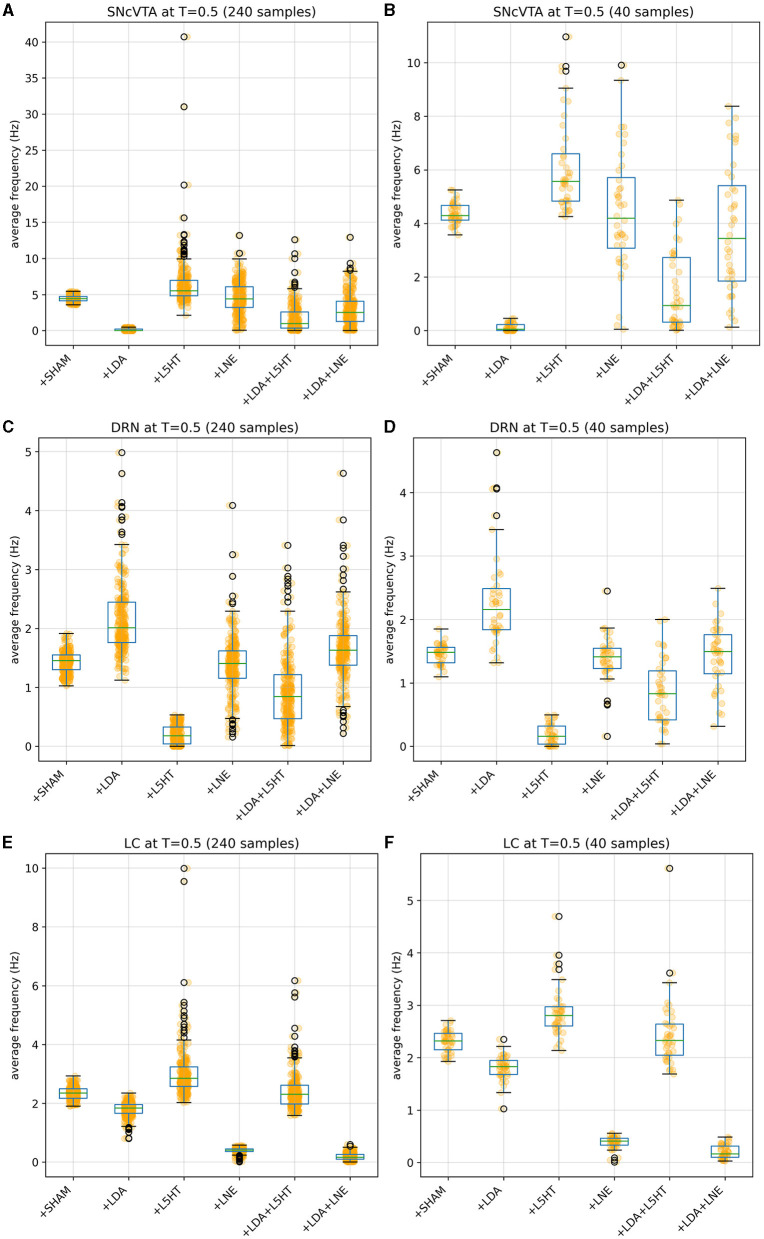
Distribution of simulated equilibrium points for the SNcVTA **(A)**, DRN **(C)**, and LC **(E)** regions across the entire synthetic population. On the right, the same simulated equilibrium points for SNcVTA **(B)**, DRN **(D)**, and LC **(F)** are displayed within separate bins, each containing 40 samples.

Figures 4, 5 give more insight in the behavior of each modeled area, also offering a comparison between the distributions over the whole population (which unfortunately could never be measured *in vivo*) and the more realistic sampled population distributions. The left graphs show distributions over the whole population (synthetic result), while on the right populations are sliced to have each subject in only one group (as in Figure 3). This latter case reproduces real laboratory conditions where one subject can only be measured once and hence belongs to one group only. Values for SHAM, LDA, L5HT, and LNE are fitted exactly on the required value, while the respective combinations are instead predictions of the model, whose values are only range-constrained with the rules defined in *F*^*COMB*^ (see Equation 55 in Appendix 2.5).

### 4.1 Sensitivity analysis

We used the computational model to gain insights into the critical parameters that govern the activity of simulated brain regions. Specifically, we conducted a sensitivity analysis for each simulated brain area in relation to the various model parameters. Each parameter can be varied independently (within some acceptable range) to record its effects on the simulated brain areas. Similar observations can be replicated for all individuals of the available population, and the average excursion of each area can then be compared with the average excursion of the parameter to infer a sort of “parameter importance”. Below, we briefly discuss the mathematical formalism used to conduct the sensitivity analysis (Equations 19, 20).

In particular, for each individual of the population and for each parameter, we:

- Vary the parameter around its original value ±50% in 100 uniform steps: if *v* is the parameter value for individual $S_{i}^{SHAM}$, we produce the set $V_{param,i}=\left\{\left(\frac{x}{99}+\frac{97}{198}\right)v\right\}\subseteq \left[0.5v,1.5v\right]$, *i* = 1, ..., 100. We therefore have a *V*_*param, i*_ set for each parameter of each individual.
- Simulate the model using each value in *V*_*param, i*_ and save the final value for each brain area. If the simulation stops early (hence the simulation diverged or reached physically impossible states), the result is discarded and the parameter value removed from *V*_*param, i*_. For each parameter and each individual, we therefore obtain six sets, one for each brain area, which we call $A_{param,i}^{area}$.

The sets are then joined across the population:


(19) $$\begin{array}{l} A_{param}^{area}=\underset{i}{⋃}A_{param,i}^{area},\;V_{param}=\underset{i}{⋃}V_{param,i} \end{array}$$

where *param*∈*S*^*SHAM*^ is one of the free parameters as defined in Section 3.1, *area* is one of the six brain areas {*GP, StrD*1, *StrD*2, *SNcVTA, DRN, LC*}, and *i* points to the *i*−th subject in the population.

A sensitivity index is then computed for each area by scaling both *V* and *A* by their respective median values and dividing the standard deviations:


(20) $$\begin{array}{l} I_{param,area}=\frac{\text{std}\left(A_{param}^{area}/\text{median}\left(A_{param}^{area}\right)\right)}{\text{std}\left(V_{param}/\text{median}\left(V_{param}\right)\right)} \end{array}$$

**Figure 6:**
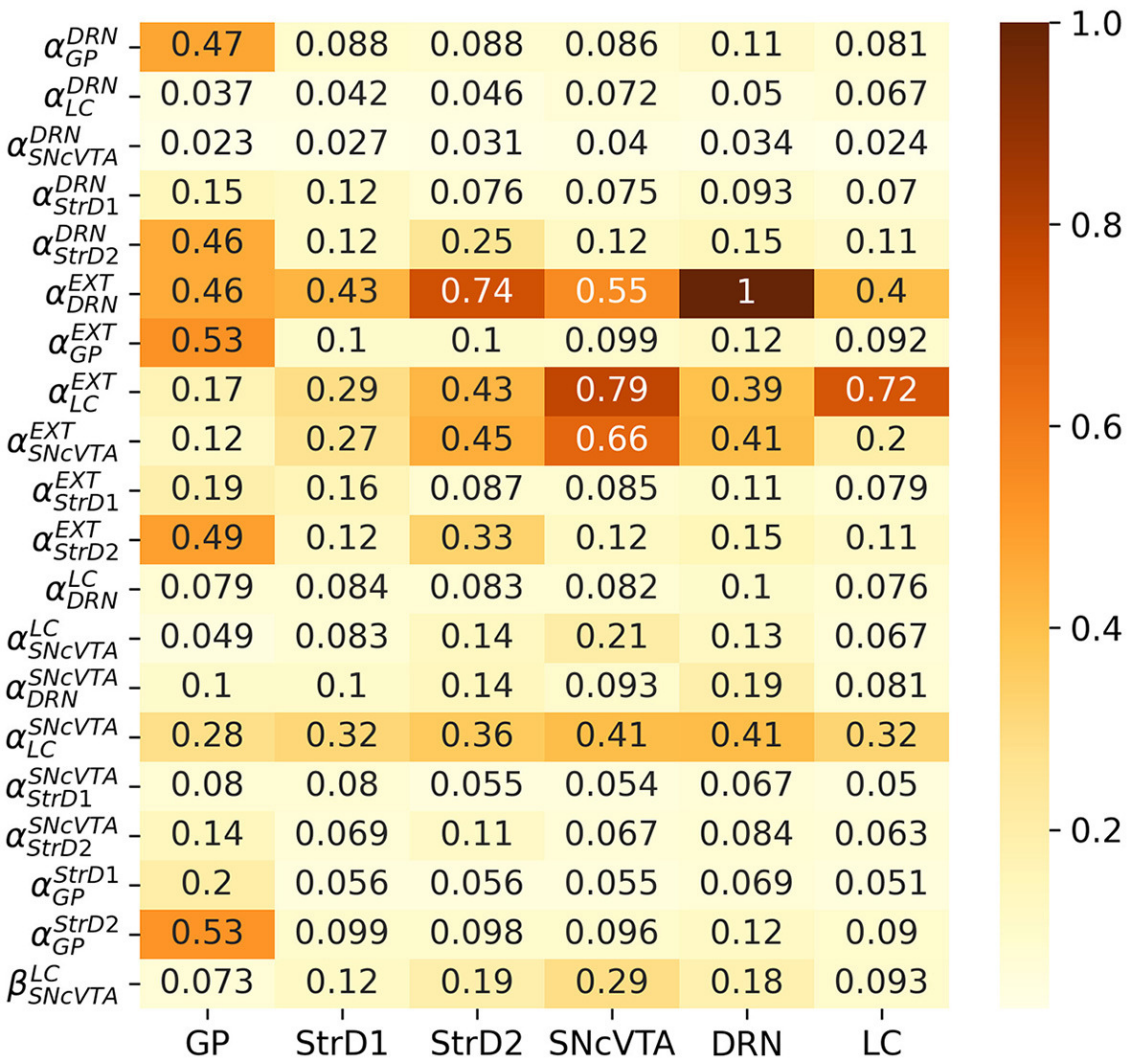
Comparative sensitivity of each area to individual parameters among healthy subjects. A higher value denotes a more pronounced impact (in absolute magnitude) of a parameter on the activation frequency of an area. Values are normalized to unity, highlighting the relative magnitude of effects obtained from each parameter.

*I*_*param, area*_ can naturally be seen as a *sensitivity matrix*, with one column per area and one row per free parameter. As a last step, *I*_*param, area*_ is normalized with respect to its maximum value. Figure 6 shows the computed sensitivity matrix for the entire fitted population in the SHAM case; a value of 1 indicates the maximum measured sensibility, while a value of 0 would mean that a particular parameter has no effect on that area. It is evident from the matrix that all the areas are relatively sensitive to changes in noradrenalinergic balance (external activation of LC), and also, even if in a somewhat lesser extent, to changes in the serotonergic balance (external activation of DRN). It is therefore reasonable to expect the stimulation of LC and/or DRN to produce changes in the activation of all areas.

### 4.2 Statistical analysis

Before performing the statistical analysis, all data were checked to verify normal distribution applying D'Agostino and Pearson's normality test, as provided by the scipy package. When appropriate, one-way ANOVA by *post-hoc* test using Tukey's honestly significant difference (HSD) (also as provided by the scipy package) was performed. Histograms are annotated according to the *p*-value of the HSD test as follows: ^****^ ≤ 0.0001 < ^***^ ≤ 0.001 < ^**^ ≤ 0.01 < ^*^ ≤ 0.05.

### 4.3 Possible roads to a treatment

We used the model that accurately replicates existing data to forecast potential outcomes in scenarios where experimental measurements have not yet been conducted. Our primary emphasis was on exploring alternative treatments centered around the manipulation of monoamines. The robustness of the model data reproduction, while rigorously adhering to stability criteria, bolstered the strength of our predictions.

**Figure 7:**
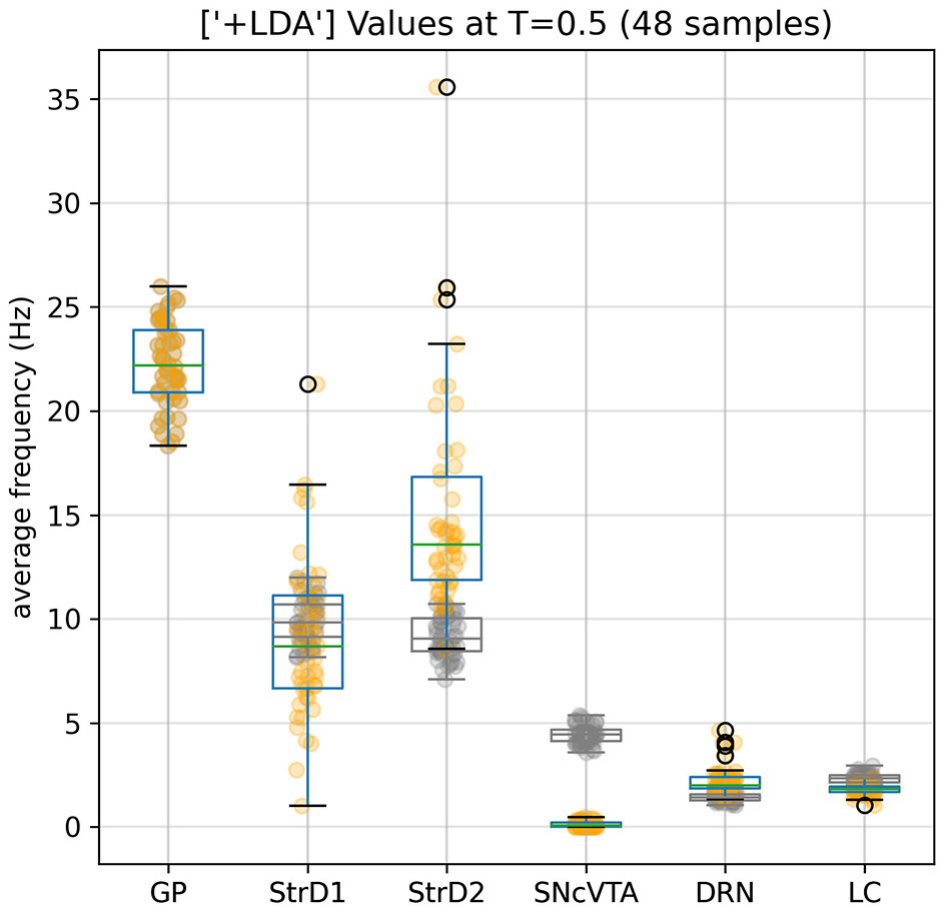
Effects of the dopaminergic (Parkinsonian) lesion to SNcVTA and LC induced by LDA. SNcVTA activation, and consequently the production of dopamine, is drastically lowered compared to the reference (gray) levels. LC activity is also lowered to 80% of its SHAM value. GP values remain unaltered as constrained by the fitness function; SNcVTA and LC are also subject to a softer constraint (see Appendix 2.3).

**Figure 8:**
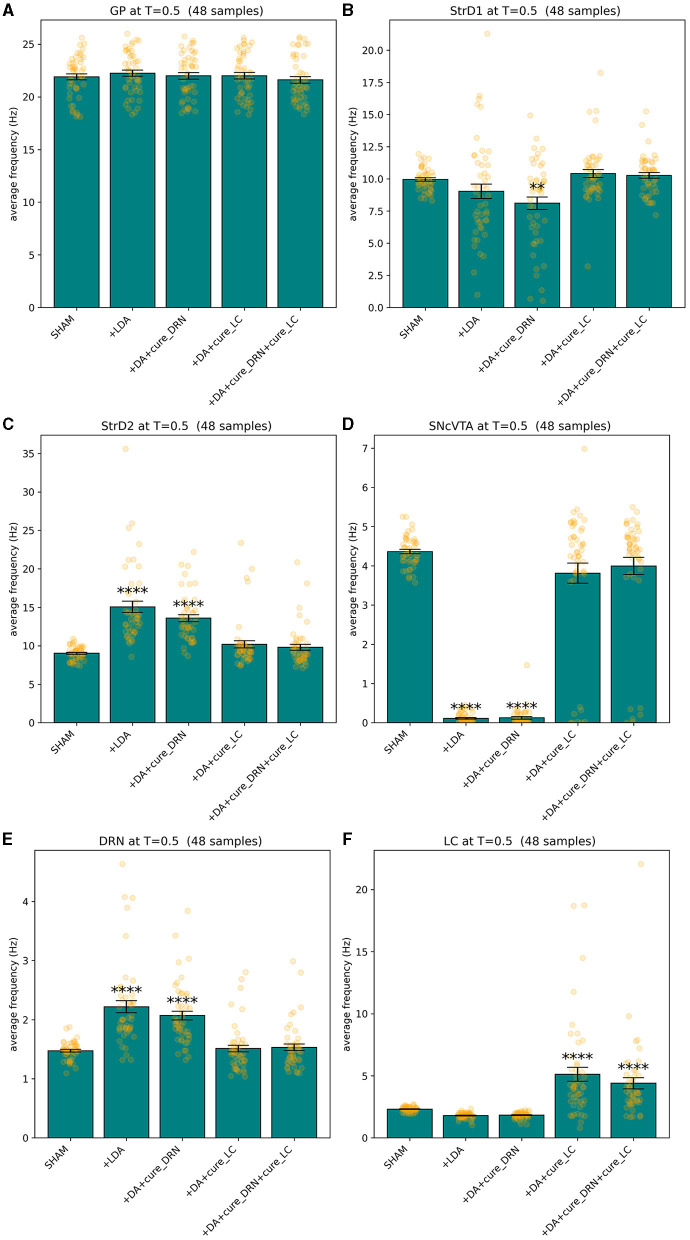
Lesion and treated values for GP **(A)**, StrD1 **(B)**, StrD2 **(C)**, SNcVTA **(D)**, DRN **(E)**, and LC **(F)**. A statistically significant boost of LC average activity (and hence of noradrenaline levels) can restore the activity (and hence monoamine production levels) of all the areas that were significatively impacted by LDA to SHAM levels. ^**^p ≤ 0.01 and ^****^p ≤ 0.0001.

Comparing the brain area activation level distributions in the SHAM to the LDA groups (Figures 7, 8), it is evident that the dopaminergic depletion also inhibits DRN and hence provokes a statistically significant serotonergic depletion. This behavior is compatible with the serotonin measurements reported in Delaville et al. (2012). The administration of LDA, however, does not alter significatively the behavior of LC (and hence noradrenaline production). In the context of dopaminergic depletion, particularly due to a lesion in the SNcVTA caused by LDA, we engage in simulations aimed at exploring strategies to alleviate activity dysfunctions by targeting alternative monoaminergic circuits.

According to the model schema in Figure 1, dopaminergic levels can potentially be altered in two ways:

1. Externally stimulate the LC to change its production of noradrenaline.
2. Externally stimulate the DRN change its production of serotonin.

The stimulation could either be chemical, by providing the area of the precursors needed to generate monoamines, or electrical, to artificially alter the average firing rate of the neurons from that area (and hence producing and projecting more monoamines to the areas which receive projections from the stimulated one). According to the sensitivity matrix in Figure 6, although, it is reasonable to expect LC stimulation to be strongly influential on dopamine levels, but DRN stimulation should have a smaller effect on the activation of the SNcVTA and strong side effects instead, which would not be compatible with a successful treatment.

#### 4.3.1 Treatment optimization

Whether the stimulation of LC, DRN, or both could potentially restore healthy levels of brain areas in depleted subjects can be verified through the optimization of a subset of the parameters of our model. In particular, we can try to optimize the external stimulation parameter of LC, DRN, or both in the LDA version of our simulated subjects. First of all, we need to extend a subject set of parameters *S*_*i*_, as previously defined in Section 3.1, with three new subsets of parameters, namely:

- LDA + cLC with free parameters $\alpha_{\text{LC}}^{\text{ext}}$ and corresponding fitness measure *F*^*cLC*^
- LDA + cDRN: $\alpha_{\text{DRV}}^{\text{ext}}$ on *F*^*cDRN*^
- LDA + cCOMB: $\alpha_{\text{LC}}^{\text{ext}}$,$\alpha_{\text{DRV}}^{\text{ext}}$ on *F*^*cCOMB*^

The corresponding model matrices *A, C*, **b** are constructed by using as base the LDA (hence, dopamine depleted) set of parameters for a subject and leaves as the only free parameters the external stimulation of the areas being tested. The three populations need to have different fitness measures because they each stimulate a different area to simulate the treatment. The stimulated area must of course be ignored by the respective fitness measure. Let us examine in detail the measure *F*^*cLC*^. Similarly to the composed fitness measure described in Appendix 2.1 for the healthy model, this measure is defined as the composition of the following measures:

- The mean square error of the area activation value, one measure per area, as defined for the SHAM case in Equation 38 in Appendix 2.2, but excluding the area being stimulated (in this case, excluding LC).
- A parameter constraint similar to the one defined in Equation 56 in Appendix 2.6, but this time used to enforce the external stimulus parameter to be equal or greater than the original once (hence forcing the optimization to choose a stimulation rather than an inhibition). In particular, the component is defined as in Equation 21:

(21) $$\begin{array}{l} f_{cLC}^{PAR}=\frac{1}{1+max\left(0,S^{SHAM}-S^{cLC}\right)}. \end{array}$$

- An asymptotic stability constraint as defined in Equation 58 in Appendix 2.7, where of course Ã is constructed using the current parameters subset *S*^*LDA*+*cLC*^.

The fitness measures *F*^*cDRN*^ and *F*^*cCOMB*^ are of course constructed in an analogous way. In the latter case, mean square errors for both stimulated areas are ignored in the measure. The optimization is finally performed independently on all subjects of the three groups using the same algorithm described in Section 3.6, including the outer optimization cycles.

#### 4.3.2 Treatment efficacy

**Figure 9:**
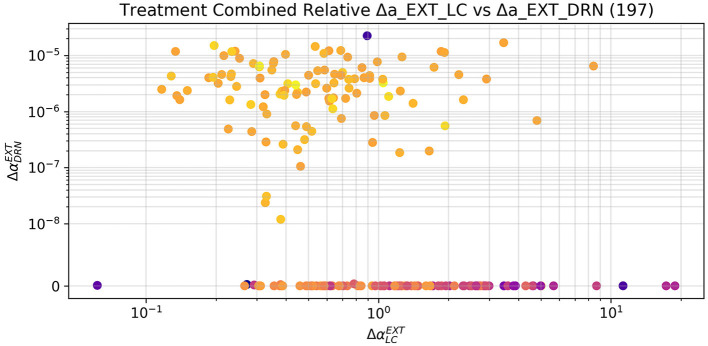
Relative increment applied by the optimizer to the external stimulation parameter of LC and DRN in the combined case, limited to the 197 subjects that were successfully treated. The increments to the DRN stimulation are several orders of magnitude smaller than the ones applied to LC. The sole stimulation of DRN is not a viable treatment. The small changes applied to the DRN stimulation by the optimizer may therefore have contributed to a numerically better solution, which is, however, not substantially different from the one obtain by the sole stimulation of LC. The subjects which did not reach a fitness of 5 (and hence are not to be considered successfully treated) have been excluded from this plot. The color scale gives an indication of the final fitness reached by the optimizer, blue is the lowest and yellow the highest.

The optimizer could successfully restore healthy levels of the measured areas in the vast majority of subjects by stimulating LC or both LC and DRN, but it never succeeded by only stimulating DRN. In particular, there was convergence in 197 subjects (which we refer here as responders) and did not find a satisfactory solution in the remaining 43 subjects (which we refer here as non-responders). Figure 9 shows that in the combined treatment, which obtained very similar results to the stimulation of LC alone, the relative increment to the external stimulation parameter of DRN is in fact several orders of magnitude smaller than the one applied to the corresponding parameter for LC. We can therefore assume that while the combined stimulation may have resulted in a slightly better fitness from the purely numerical perspective, DRN stimulation is indeed not useful as a treatment also in combination to LC stimulation.

Figure 8 provides compelling evidence of the profound impact of statistically significant LC stimulation in reinstating the equilibrium of serotonin and dopamine levels, as reflected in the activation levels of DRN and SNcVTA, respectively, within the population of LDA subjects.

**Figure 10:**
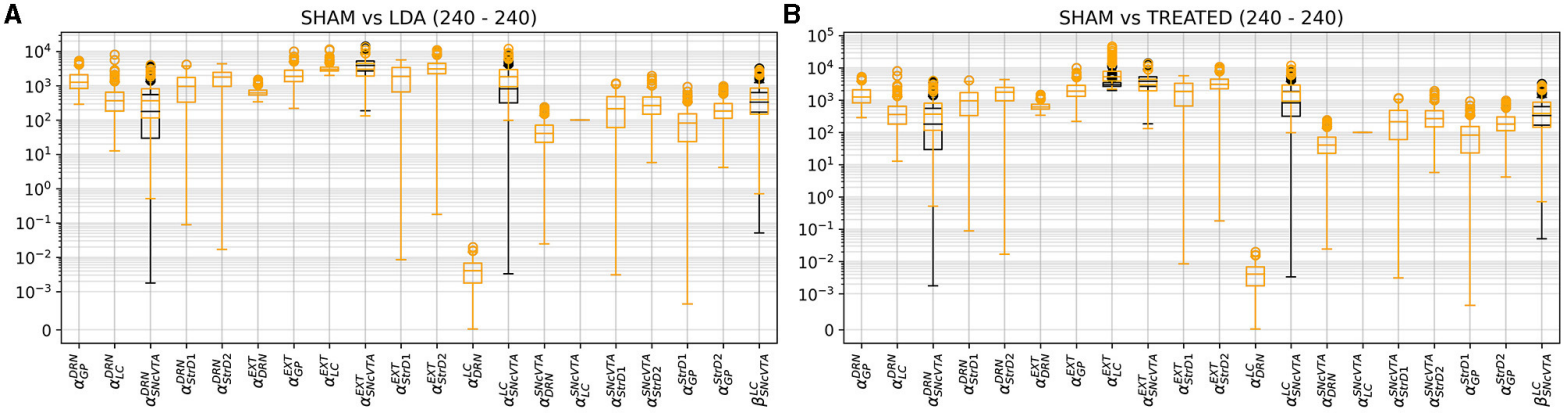
Comparison of parameter distribution among model subjects: SHAM subjects represented in black, while LDA subjects under two conditions, either LDA **(A)** or treated **(B)**, depicted in orange. As outlined in Section 2.3, only four parameters, impacting the SNcVTA equation, undergo alteration in the LDA scenario compared to SHAM. In addition, the treatment induces changes in external stimulation to LC and DRN.

**Figure 11:**
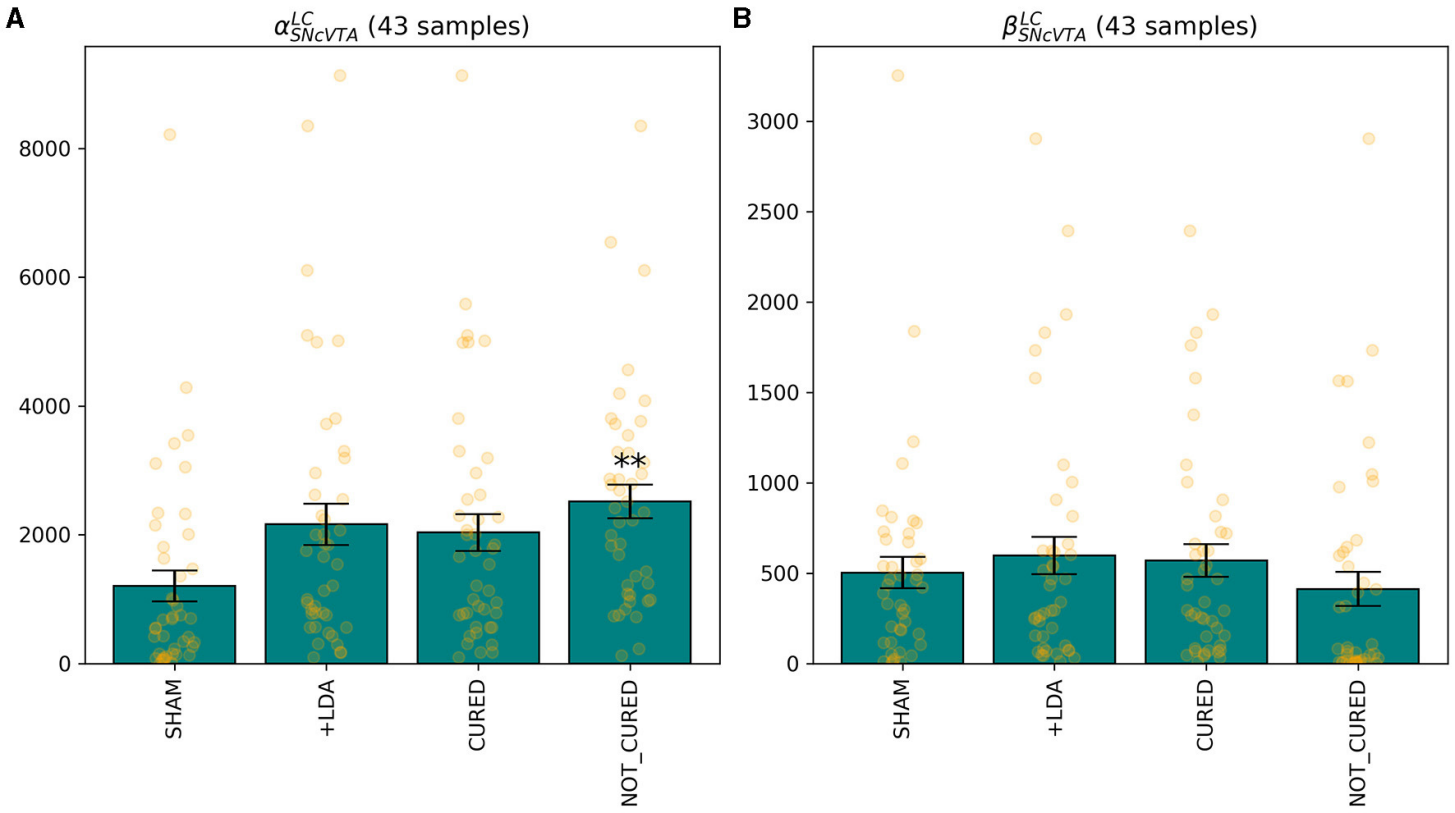
The only feature that differentiates, albeit with low significance, individuals for which it was possible to find a treatment stimulating either LC or DRN is the sensitivity of SNcVTA to noradrenaline from LC, for both the linear **(A)** and non-linear **(B)** terms. ^**^p ≤ 0.01.

**Figure 12:**
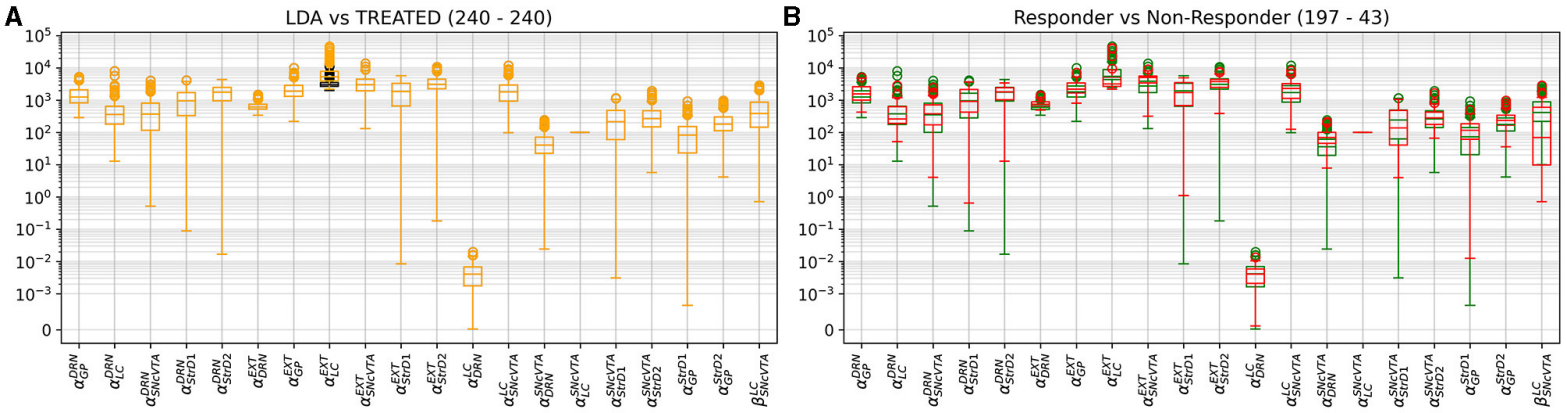
Distribution comparison of model parameters. In **(A)**, a distribution comparison of model parameters in LDA subjects relative to treated subjects is presented. In **(B)**, the parameter distribution of treated subjects is depicted, distinguishing between those successfully cured (green) and those who did not attain the desired fitness (red). Notably, among the non-responsive subjects, one exhibited reduced sensitivity of SNcVTA to noradrenaline.

Figures 10–12 illustrate the changes of distributions in the parameter space lesion and the subsequent treatment. The right side of Figure 12 highlights the differences in parameter distributions between the subjects that have been successfully treated (in green), and the ones whose levels could not be successfully restored (in red). None of the parameters of the responder subjects are significantly different from the one of the non-responder ones. The only parameter that shows a small significance difference is the sensitivity of SNcVTA toward noradrenaline from LC, as is shown in Figure 11. However, the spread of the distribution of that parameter is very large, and the value of that specific parameter alone is not useful for predicting if a subject is a responder or not. An accurate statistical study of the parameter space would be necessary to determine whether a particular combination of parameters could be used for predicting the curability of a subject, but this lies outside the scope of this study.

Figure 12 also shows the parameter space comparison of responder (labeled “cured") and non-responder (labeled “not cured") individuals for all parameters. The treatment modifies the external stimulation to LC and DRN but is otherwise identical to the LDA case. This result agrees with Figure 9 demonstrating that there is a very small shift in the DRN stimulus distribution which is not appreciable in Figure 12.

## 5 Discussion

PD is a global health concern, impacting an estimated 10 million individuals worldwide (Balestrino and Schapira, 2020). Regrettably, a definitive treatment for PD remains elusive. However, there is optimism in the research community as numerous novel drugs are presently undergoing clinical trials. One of the pivotal facets of PD is its intricate association with various neural circuits, resulting in consequential alterations in the brain neurochemistry (Obeso et al., 2010; Caligiore et al., 2016, 2017; Helmich et al., 2018). Our research endeavor confronts the challenge of exploring the interplay among these distinct circuits and how the monoamine system adapts during the progression of this condition.

To tackle this challenge, we employed a bio-constrained differential equation model of PD. This model enabled us to investigate how different brain regions respond to the individual or combined depletion of dopamine (DA), serotonin (5-HT), and noradrenaline (NA). Initially, we harnessed the model to replicate data obtained from Delaville et al. (2012). The simulation results remarkably mirrored the observed alterations in the firing activity of the globus pallidus (GP) following 5-HT lesions in both control and dopamine-depleted rats. Notably, the model also yielded predictions of other deviations in firing activity within brain regions not examined in the original experimental setup involving rats. Ultimately, our model served as a valuable tool to simulate the effects of prospective therapies aimed at restoring the activity in the lesioned regions. To model the impairment of DA function associated with PD, we implemented a reduction in the activity of the SNcVTA (Berretta et al., 2005). As a result, we have a dysregulation of the baseline activity within the specific brain regions we examined. Notably, DRN exhibited a notable increase in firing activity (see Figures 3, 5) in line with the literature (Kaya et al., 2008; Zhang et al., 2018; Caligiore et al., 2021). The DRN intrinsic excitability (Prinz et al., 2013) projections back from the SNcVTA to the DRN (Kalen et al., 1988; Peyron et al., 1996; Kitahama et al., 2000) might support this effect. In addition, dopamine agonist increases excitation on DRN serotonergic neurons (Haj-Dahmane, 2001; Martın-Ruiz et al., 2001). However, there is also literature supporting that there is no change in the electrical activity of serotonergic-like neurons following L-Dopa injection (Miguelez et al., 2011, 2016), that lesion in VTA could lead to a reduction of serotonin in DRN, and that supports a DRN activity reduction after a dopaminergic lesion (Furlanetti et al., 2016). The different results showed in DRN firing activity showed in this literature might depend on several factors such as the type of lesion (unilateral or bilateral), the site of lesion, and time of recording after lesion. Moreover, we excluded from our model several brain regions that might affect DRN regulation, for example, the medial prefrontal cortex and subthalamic nucleus playing a critical role in the inhibition of DRN (Celada et al., 2001; Temel et al., 2007; Hartung et al., 2011). It could be possible that during the initial stages of the disease, the DRN activity increases, while it decreases with the disease progression. The inversed u-shape followed by the DRN activity with the PD progression could be similar to that followed by LC activity during PD and AD progression (Caligiore et al., 2020, 2022; O'Callaghan et al., 2021; Ye et al., 2023). Further research could confirm or refute this hypothesis.

The simulations run with the model show a reduction in striatal D1 activity and an increase in striatal D2 activity (see Figures 3, 4). This result agrees with the literature about hypokinetic Parkinsonian syndrome due to the activity dysregulation of the two populations of medium spiny neurons (MSNs). Dopamine D1 receptor-expressing MSNs (direct) become hypoactive, whereas dopamine D2 receptor-expressing MSNs (indirect) become hyperactive. The global effect is the excitation of the direct pathway and the inhibition of the indirect pathway, both conditions necessary for correctly managing motor output (Albin et al., 1989; Mallet et al., 2006; Parker et al., 2018; Caligiore et al., 2019).

The simulations also show a reduction of LC activity after dopaminergic depletion (Figures 3, 4). The DA excitatory effect on LC reproduced in our model could explain this result. However, the literature supports both decrease and increase in LC, depending on the PD animal models used for experiments (Ranjbar-Slamloo and Fazlali, 2020). Thus, future *in vivo* studies should confirm or refute the model result. The serotonin lesions allow reproduction of the GP firing activity decrease reported in the target experiment (Delaville et al., 2012). Moreover, the lesioned model shows an increase in SNcVTA activity (Figures 3, 5). The 5-HT inhibitory effect on dopaminergic neurons reproduced in our model could explain this result. However, future studies are required to confirm these data. Indeed, 5-HT could modulate SNpc and VTA DA neurons oppositely (Gervais and Rouillard, 2000).

### 5.1 Exploring potential monoamine-based therapies

Levodopa is the most common medication used in PD. However, this drug has a wide range of adverse effects, most notably motor fluctuations and dyskinesias (for a rev see Lang and Obeso, 2004). The discovery of alternative treatment that not only targets dopaminergic system but also noradrenergic or serotonergic system is a big challenge (Politis and Niccolini, 2015; Wilson et al., 2019; Caligiore et al., 2021, 2022). Figures 9–12 collectively indicate that the restoration of activity in the LC is sufficient to restore the regions under examination, while the DRN does not yield the same effect. This observation is consistent with current theories that underscore the central role of the LC in the progression of PD, particularly in relation to the early-stage emergence of non-motor symptoms (Bjerkén et al., 2019; Butkovich et al., 2020). Moreover, there is literature supporting the notion that the overexpression of specific transcription factors directly within the LC can effectively restore noradrenergic function and facilitate the recovery of the dopaminergic system (Cui et al., 2021). Notably, in the context of human studies, mounting evidence underscores the fact that LC degeneration can manifest at an earlier stage and with greater severity compared to the SNcVTA (German et al., 1992; Del Tredici et al., 2002; Rommelfanger and Weinshenker, 2007; Vermeiren and Deyn, 2017). Taken together, these findings strongly suggest that the LC may play a pivotal role in the emergence and progression of PD and present an intriguing avenue for therapeutic interventions targeting both the dopaminergic and noradrenergic systems.

The feasibility of this discovery is not straightforward. One plausible explanation for the lack of productivity of novel drugs targeting monoaminergic function could be the complexity of the brain neurotransmitter systems. While these systems play crucial roles in regulating mood and cognition, their functions are highly interconnected and often involve feedback loops and compensatory mechanisms. It is possible that the drugs developed to target these systems may inadvertently disrupt the delicate balance of neurotransmitter activity, leading to unintended side effects or diminishing efficacy over time. In addition, the existing drugs may primarily target specific receptor subtypes within the noradrenergic systems, leaving other potential therapeutic targets unexplored. This limited scope could hinder the development of more effective treatments (Tan et al., 2022; Pardo-Moreno et al., 2023). Finally, while the model suggests promising alternative treatments targeting the LC and DRN, it is crucial to consider the potential side effects associated with these approaches. Given the critical roles these regions play in regulating mood, arousal, and autonomic functions, interventions could lead to increased anxiety, sleep disturbances, and cardiovascular issues. These potential side effects highlight the need for comprehensive experimental validation and clinical studies to assess the safety and efficacy of these alternative treatments. Addressing these concerns will be essential for translating our model predictions into practical therapeutic strategies.

## 6 Conclusion

This article presents a novel system-level computational model that, for the first time, explores the roles of three important monoamines (dopamine, serotonin, and noradrenaline) in a PD animal model. The model assumes a direct relationship between reduced activity in specific brain areas (SNcVTA, DRN, and LC) and decreased neurotransmitter levels (DA, 5-HT, and NE) in target brain regions. In addition, it simulates VTA and SNc together and excludes some potentially relevant areas, such as the prefrontal and motor cortices, thalamus, and substantia nigra pars reticulata. Despite these simplifications, the model successfully reproduces some data on combined monoamine depletion, generates predictions regarding changes in other unexplored brain regions, suggesting avenues for further investigation, and highlights the potential efficacy of alternative treatments targeting the locus coeruleus and dorsal raphe nucleus. This suggests that the model captures crucial aspects of the interactions between the considered areas. Furthermore, it may also imply that the aspects not yet modeled might only play secondary roles in the system behaviors.

We do not subscribe to this belief. Our plan is to expand the model to incorporate more areas involved in PD (Dirkx et al., 2017; Helmich et al., 2018), to more accurately delineate macro-areas like SNc/VTA (Ledonne et al., 2023), and to include additional interaction paths. The upgraded versions of the model may be well-suited for delving into more mechanisms, such as those elucidating the effects of dopamine receptor activation (Mailman et al., 2021; Lewis et al., 2023) or exploring monoamine interactions within various brain regions (Oh et al., 2022). At this stage, the model represents an initial effort to employ a system-level computational approach to address the intricacies of PD as a systemic disease, with a focus on the interplay of monoamines. It serves as an initial attempt to demonstrate the feasibility of this approach, employing a rigorous mathematical framework specifically, the study of stability, a seldom-used approach in computational neuroscience. The results obtained through the model are preliminary, and their effectiveness requires further validation.

In this respect, a series of experimental approaches could validate the model predictions. *In vivo*, neurochemical assessments using microdialysis in PD animal models could measure extracellular levels of DA, NE, and 5-HT in the basal ganglia. Pharmacological manipulations could selectively alter NE and 5-HT levels, observing the effects on PD symptoms and neurochemical dynamics to validate the model predictions about these systems. Neuroimaging studies using functional magnetic resonance imaging (fMRI) and positron emission tomography (PET) can assess functional connectivity and receptor density changes in brain regions highlighted by the model. This analysis could help validate the predicted alterations in neuromodulator levels and activity patterns. In addition, analyzing functional connectivity using high-density electroencephalography (HD-EEG) could validate the model on a macroscale by measuring changes in band-specific cortico-cortical and subcortico-cortical connectivity before and after manipulations of the monoaminergic system (Conti et al., 2023). Moreover, electrophysiological recordings of neuronal activity in relevant brain regions before and after such manipulations could validate the model's predictions regarding neural circuitry alterations.

The model proposed here could enhance our comprehension of interactions between brain regions in both normal and pathological conditions, potentially aiding in the restoration of damaged brain regions to reestablish balance. The model could be adapted to study other neurodegenerative and neuropsychiatric disorders involving similar monoaminergic systems. For instance, there is increasing evidence of the critical role of monoamines in AD (Caligiore et al., 2022) and multiple sclerosis (Carandini et al., 2021). Adapting the model to these disorders would involve modifying the parameters to reflect the specific pathophysiological mechanisms and neurochemical dynamics unique to each condition. Future research could address these points.

## Data Availability

The datasets presented in this study can be found in online repositories. The names of the repository/repositories and accession number(s) can be found at: https://github.com/WohthaN/Simulating_noradrenaline_and_serotonin_depletions_in_parkinson.

## References

[B1] Aarsland D. Bronnick K. Larsen J. P. Tysnes O. B. Alves G. (2009). Cognitive impairment in incident, untreated Parkinson disease: the Norwegian ParkWest Study. Neurology 72, 1121–1126. 10.1212/01.wnl.0000338632.00552.cb 19020293

[B2] Albin R. L. Young A. B. Penney J. B. (1989). The functional anatomy of basal ganglia disorders. Trends Neurosci. 12, 366–375. 10.1016/0166-2236(89)90074-X 2479133

[B3] Antonini A. Emmi A. Campagnolo M. (2023). Beyond the dopaminergic system: lessons learned from levodopa resistant symptoms in Parkinson's disease. Mov. Disord. Clin. Pract. 10:S50. 10.1002/mdc3.13786 37637981 PMC10448140

[B4] Balestrino R. Schapira A. H. (2020). Parkinson disease. Eur. J. Neurol. 27, 27–42. 10.1111/ene.14108 31631455

[B5] Berretta N. Freestone P. S. Guatteo E. de Castro D. Geracitano R. Bernardi J. (2005). Acute effects of 6-hydroxydopamine on dopaminergic neurons of the rat substantia nigra pars compacta *in vitro*. Neurotoxicology 26, 869–881. 10.1016/j.neuro.2005.01.014 15890406

[B6] Bjerkén S. Persson R. S. Barkander A. Karalija N. Pelegrina-Hidalgo N. Gerhardt G. A.. (2019). Noradrenaline is crucial for the substantia nigra dopaminergic cell maintenance. Neurochem. Int. 131:104551. 10.1016/j.neuint.2019.104551 31542295

[B7] Butcher J. C. (2016). Numerical Methods for Ordinary Differential Equations, 3rd Edn. Chichester: Wiley Blackwell. 10.1002/9781119121534

[B8] Butkovich L. M. Houser M. C. Chalermpalanupap T. Porter-Stransky K. A. Iannitelli A. F. Boles J. S.. (2020). Transgenic mice expressing human α-synuclein in noradrenergic neurons develop locus coeruleus pathology and nonmotor features of Parkinson's disease. J. Neurosci. 40, 7559–7576. 10.1523/JNEUROSCI.1468-19.2020 32868457 PMC7511194

[B9] Byrne G. D. Hindmarsh A. C. (1975). A polyalgorithm for the numerical solution of ordinary differential equations. ACM Trans. Math. Softw. 1, 71–96. 10.1145/355626.355636

[B10] Caligiore D. Giocondo F. Silvetti M. (2022). The Neurodegenerative Elderly Syndrome (NES) hypothesis: Alzheimer and Parkinson are two faces of the same disease. IBRO Neurosci. Rep. 13, 330–343. 10.1016/j.ibneur.2022.09.007 36247524 PMC9554826

[B11] Caligiore D. Helmich R. C. Hallett M. Moustafa A. A. Timmermann L. Toni I.. (2016). Parkinson's disease as a system-level disorder. NPJ Parkinson's Dis. 2:16025. 10.1038/npjparkd.2016.25 28725705 PMC5516580

[B12] Caligiore D. Mannella F. Baldassarre G. (2019). Different dopaminergic dysfunctions underlying parkinsonian akinesia and tremor. Front. Neurosci. 13:550. 10.3389/fnins.2019.00550 31191237 PMC6549580

[B13] Caligiore D. Montedori F. Buscaglione S. Capirchio A. (2021). Increasing serotonin to reduce parkinsonian tremor. Front. Syst. Neurosci. 15:682990. 10.3389/fnsys.2021.682990 34354572 PMC8331097

[B14] Caligiore D. Pezzulo G. Baldassarre G. Bostan A. C. Strick P. L. Doya K.. (2017). Consensus paper: towards a systems-level view of cerebellar function: the interplay between cerebellum, Basal Ganglia, and Cortex. Cerebellum 16, 203–229. 10.1007/s12311-016-0763-3 26873754 PMC5243918

[B15] Caligiore D. Silvetti M. D'Amelio M. Puglisi-Allegra S. Baldassarre G. (2020). Computational modeling of catecholamines dysfunction in Alzheimer's disease at pre-plaque stage. J. Alzheimers Dis. 77, 275–290. 10.3233/JAD-200276 32741822 PMC7592658

[B16] Carandini T. Cercignani M. Galimberti D. Scarpini E. Bozzali M. (2021). The distinct roles of monoamines in multiple sclerosis: a bridge between the immune and nervous systems? Brain Behav. Immun. 94, 381–391. 10.1016/j.bbi.2021.02.030 33662501

[B17] Castela I. Casado-Polanco R. Rubio Y. V.-W. da Silva J. A. Marquez R. Pro J.. (2023). Selective activation of striatal indirect pathway suppresses levodopa induced-dyskinesias. Neurobiol. Dis. 176:105930. 10.1016/j.nbd.2022.105930 36414182

[B18] Celada P. Puig M. V. Casanovas J. M. Guillazo G. Artigas F. (2001). Control of dorsal raphe serotonergic neurons by the medial prefrontal cortex: involvement of serotonin-1a, gaba_*a*_, and glutamate receptors. J. Neurosci. 21, 9917–9929. 10.1523/JNEUROSCI.21-24-09917.2001 11739599 PMC6763042

[B19] Chen L. Yung K. Chan Y. Yung W. (2008). 5-ht excites globus pallidus neurons by multiple receptor mechanisms. Neuroscience 151, 439–451. 10.1016/j.neuroscience.2007.11.003 18082329

[B20] Cohen E. Bay A. A. Ni L. Hackney M. E. (2022). Apathy-related symptoms appear early in Parkinson's disease. Healthcare 10:91. 10.3390/healthcare10010091 35052255 PMC8775593

[B21] Connolly B. S. Lang A. E. (2014). Pharmacological treatment of Parkinson disease: a review. J. Am. Med. Assoc. 311, 1670–1683. 10.1001/jama.2014.3654 24756517

[B22] Conti M. Guerra A. Pierantozzi M. Bovenzi R. D'Onofrio V. Simonetta C.. (2023). Band-specific altered cortical connectivity in early Parkinson's disease and its clinical correlates. Mov. Disord. 38, 2197–2208. 10.1002/mds.29615 37860930

[B23] Cui K. Yang F. Tufan T. Raza M. U. Zhan Y. Fan Y.. (2021). Restoration of noradrenergic function in Parkinson's disease model mice. ASN Neuro 13:175909142110097. 10.1177/17590914211009730 33940943 PMC8114769

[B24] Damodaran S. Evans R. C. Blackwell K. T. (2014). Synchronized firing of fast-spiking interneurons is critical to maintain balanced firing between direct and indirect pathway neurons of the striatum. J. Neurophysiol. 111, 836–848. 10.1152/jn.00382.2013 24304860 PMC3921391

[B25] Deister C. A. Chan C. S. Surmeier D. J. Wilson C. J. (2009). Calcium-activated sk channels influence voltage-gated ion channels to determine the precision of firing in globus pallidus neurons. J. Neurosci. 29, 8452–8461. 10.1523/JNEUROSCI.0576-09.2009 19571136 PMC3329865

[B26] Del Tredici K. Rüb U. De Vos R. A. Bohl J. R. Braak H. (2002). Where does Parkinson disease pathology begin in the brain? J. Neuropathol. Exp. Neurol. 61, 413–426. 10.1093/jnen/61.5.413 12030260

[B27] Delaville C. Chetrit J. Abdallah K. Morin S. Cardoit L. De Deurwaerdère P.. (2012). Emerging dysfunctions consequent to combined monoaminergic depletions in Parkinsonism. Neurobiol. Dis. 45, 763–773. 10.1016/j.nbd.2011.10.023 22079236

[B28] Delaville C. Deurwaerdere P. D. Benazzouz A. (2011). Noradrenaline and Parkinson's disease. Front. Syst. Neurosci. 5:31. 10.3389/fnsys.2011.00031 21647359 PMC3103977

[B29] Deutch A. Y. Goldstein M. Roth R. H. (1986). Activation of the locus coeruleus induced by selective stimulation of the ventral tegmental area. Brain Res. 363, 307–314. 10.1016/0006-8993(86)91016-4 3942901

[B30] Dirkx M. F. den Ouden H. E. Aarts E. Timmer M. H. Bloem B. R. Toni R. C. (2017). Dopamine controls Parkinson's tremor by inhibiting the cerebellar thalamus. Brain 140, 721–734. 10.1093/brain/aww331 28073788

[B31] Dovzhenok A. Rubchinsky L. L. (2012). On the origin of tremor in Parkinson's disease. PLoS ONE 7:e41598. 10.1371/journal.pone.0041598 22848541 PMC3407214

[B32] Dray A. Davies J. Oakley N. Tongroach P. Vellucci S. (1978). The dorsal and medial raphe projections to the substantia nigra in the rat: electrophysiological, biochemical and behavioural observations. Brain Res. 151, 431–442. 10.1016/0006-8993(78)91077-6 667623

[B33] Dray A. Gonye T. Oakley N. Tanner T. (1976). Evidence for the existence of a raphe projection to the substantia nigra in rat. Brain Res. 113, 45–57. 10.1016/0006-8993(76)90005-6 953733

[B34] Espay A. J. Aybek S. Carson A. Edwards M. J. Goldstein L. H. Hallett M.. (2018). Current concepts in diagnosis and treatment of functional neurological disorders. JAMA Neurol. 75:1132. 10.1001/jamaneurol.2018.1264 29868890 PMC7293766

[B35] Faivre F. Joshi A. Bezard E. Barrot M. (2019). The hidden side of Parkinson's disease: studying pain, anxiety and depression in animal models. Neurosci. Biobehav. Revi. 96, 335–352. 10.1016/j.neubiorev.2018.10.004 30365972

[B36] Favier M. Carcenac C. Savasta M. Carnicella S. (2022). Dopamine D3 receptors: a potential target to treat motivational deficits in Parkinson's disease. Curr. Top. Behav. Neurosci. 60, 109–132. 10.1007/7854_2022_316 35469394

[B37] Fornari C. Pin C. Yates J. W. Mettetal J. T. Collins T. A. (2020). Importance of stability analysis when using nonlinear semimechanistic models to describe drug-induced hematotoxicity. CPT: Pharmacometrics Syst. Pharmacol. 9, 498–508. 10.1002/psp4.12514 32453487 PMC7499189

[B38] Furlanetti L. Coenen V. Dbrssy M. (2016). Ventral tegmental area dopaminergic lesion-induced depressive phenotype in the rat is reversed by deep brain stimulation of the medial forebrain bundle. Behav. Brain Res. 299, 132–140. 10.1016/j.bbr.2015.11.036 26657994

[B39] German D. C. Manaye K. F. White C. L. Woodward D. J. McIntire D. D. Smith W. K.. (1992). Disease-specific patterns of locus coeruleus cell loss. Ann. Neurol. 32, 667–676. 10.1002/ana.410320510 1449247

[B40] Gervais J. Rouillard C. (2000). Dorsal raphe stimulation differentially modulates dopaminergic neurons in the ventral tegmental area and substantia nigra. Synapse 35, 281–291. 10.1002/(SICI)1098-2396(20000315)35:4<281::AID-SYN6>3.0.CO;2-A 10657038

[B41] Haj-Dahmane S. (2001). D_2_ -like dopamine receptor activation excites rat dorsal raphe 5-HT neurons *in vitro*. *Eur. J. Neurosci*. 14, 125–134. 10.1046/j.0953-816x.2001.01616.x 11488956

[B42] Hansen L. Witzig V. Schulz J. B. Holtbernd F. (2023). Dopaminergic treatment strategies for people with Parkinson's disease in Europe: a retrospective analysis of prism trial data. Neurol. Sci. 44, 3905–3912. 10.1007/s10072-023-06888-5 37311949 PMC10570205

[B43] Hartung H. Tan S. Steinbusch H. Temel Y. Sharp T. (2011). High-frequency stimulation of the subthalamic nucleus inhibits the firing of juxtacellular labelled 5-ht-containing neurones. Neuroscience 186, 135–145. 10.1016/j.neuroscience.2011.04.004 21515342

[B44] Helmich R. C. Hallett M. Deuschl G. Toni I. Bloem B. (2012). Cerebral causes and consequences of parkinsonian resting tremor: a tale of two circuits? Brain 135, 3206–3226. 10.1093/brain/aws023 22382359 PMC3501966

[B45] Helmich R. C. Vaillancourt D. E. Brooks D. J. (2018). The future of brain imaging in Parkinson's disease. J. Parkinsons Dis. 8, S47–S51. 10.3233/JPD-181482 30584163 PMC6311365

[B46] Hezemans F. H. Wolpe N. O'Callaghan C. Ye R. Rua C. Jones P. S.. (2022). Noradrenergic deficits contribute to apathy in Parkinson's disease through the precision of expected outcomes. PLoS Comput. Biol. 18:e1010079. 10.1371/journal.pcbi.1010079 35533200 PMC9119485

[B47] Jankovic J. (2018). Parkinson's disease tremors and serotonin. Brain 141, 624–626. 10.1093/brain/awx361 30063797

[B48] Jankovic J. Kapadia A. S. (2001). Functional decline in Parkinson's disease. Arch. Neurol. 58:1611. 10.1001/archneur.58.10.1611 11594919

[B49] Jellinger K. A. (1991). Pathology of Parkinson's disease. Mol. Chem. Neuropathol. 14, 153–197. 10.1007/BF03159935 1958262

[B50] Kalen P. Skagerberg G. Lindvall O. (1988). Projections from the ventral tegmental area and mesencephalic raphe to the dorsal raphe nucleus in the rat: evidence for a minor dopaminergic component. Exp. Brain Res. 73, 69–77. 10.1007/BF00279662 3208862

[B51] Kang Y. Kitai S. (1993). A whole cell patch-clamp study on the pacemaker potential in dopaminergic neurons of rat substantia nigra compacta. Neurosci. Res. 18, 209–221. 10.1016/0168-0102(93)90056-V 8127469

[B52] Kaya A. H. Vlamings R. Tan S. Lim L. W. Magill P. J. Steinbusch H. W.. (2008). Increased electrical and metabolic activity in the dorsal raphe nucleus of parkinsonian rats. Brain Res. 1221, 93–97. 10.1016/j.brainres.2008.05.019 18565496

[B53] Kelland M. D. Freeman A. S. Chiodo L. A. (1990). Serotonergic afferent regulation of the basic physiology and pharmacological responsiveness of nigrostriatal dopamine neurons. J. Pharmacol. Exp. Ther. 253, 803–11. 1971022

[B54] Kelland M. D. Freeman A. S. Rubin J. Chiodo L. A. (1993). Ascending afferent regulation of rat midbrain dopamine neurons. Brain Res. Bull. 31, 539–546. 10.1016/0361-9230(93)90121-Q 8495379

[B55] Kelley W. G. Peterson A. C. (2001). Difference Equations: An Introduction with Applications, 2nd Edn. San Diego, CA: Harcourt/Academic Press.

[B56] Kita H. Kita T. (2011). Role of striatum in the pause and burst generation in the globus pallidus of 6-OHDA-treated rats. Front. Syst. Neurosci. 5:42. 10.3389/fnsys.2011.00042 21713126 PMC3113166

[B57] Kitahama K. Nagatsu I. Geffard M. Maeda T. (2000). Distribution of dopamine-immunoreactive fibers in the rat brainstem. J. Chem. Neuroanat. 18, 1–9. 10.1016/S0891-0618(99)00047-2 10708914

[B58] Lakshmikantham V. Trigiante D. (2002). Theory of difference equations: numerical methods and applications. Number 251 in Monographs and textbooks in pure and applied mathematics, 2nd Edn. New York, NY: Marcel Dekker. 10.1201/9780203910290

[B59] Lang A. E. Obeso J. A. (2004). Challenges in Parkinson's disease: restoration of the nigrostriatal dopamine system is not enough. Lancet Neurol. 3, 309–316. 10.1016/S1474-4422(04)00740-9 15099546

[B60] Ledonne A. Massaro Cenere M. Paldino E. D'Angelo V. D'Addario S. L. Casadei N.. (2023). Morpho-functional changes of nigral dopamine neurons in an α-synuclein model of Parkinson's disease. Mov. Disord. 38, 256–266. 10.1002/mds.29269 36350188

[B61] Lewis M. M. Van Scoy L. J. De Jesus S. Hakun J. G. Eslinger P. J. Fernandez-Mendoza N.. (2023). Dopamine d1 agonists: first potential treatment for late-stage Parkinson's disease. Biomolecules 13:829. 10.3390/biom13050829 37238699 PMC10216182

[B62] Lin Y. Quartermain D. Dunn A. J. Weinshenker D. Stone E. A. (2008). Possible dopaminergic stimulation of locus coeruleus alpha1-adrenoceptors involved in behavioral activation. Synapse 62, 516–523. 10.1002/syn.20517 18435418 PMC2754581

[B63] Liu J. Shelkar G. P. Sarode L. P. Gawande D. Y. Zhao F. Clausen R. P.. (2021). Facilitation of glun2c-containing nmda receptors in the external globus pallidus increases firing of fast spiking neurons and improves motor function in a hemiparkinsonian mouse model. Neurobiol. Dis. 150:105254. 10.1016/j.nbd.2021.105254 33421565 PMC8063913

[B64] Liu R.-J. van den Pol A. N. Aghajanian G. K. (2002). Hypocretins (orexins) regulate serotonin neurons in the dorsal raphe nucleus by excitatory direct and inhibitory indirect actions. J. Neurosci. 22, 9453–9464. 10.1523/JNEUROSCI.22-21-09453.2002 12417670 PMC6758063

[B65] Mailman R. B. Yang Y. Huang X. (2021). D1, not d2, dopamine receptor activation dramatically improves mptp-induced parkinsonism unresponsive to levodopa. Eur. J. Pharmacol. 892:173760. 10.1016/j.ejphar.2020.173760 33279520 PMC7861126

[B66] Mallet N. Ballion B. Le Moine C. Gonon F. (2006). Cortical inputs and gaba interneurons imbalance projection neurons in the striatum of parkinsonian rats. J. Neurosci. 26, 3875–3884. 10.1523/JNEUROSCI.4439-05.2006 16597742 PMC6674115

[B67] Marras C. Chaudhuri K. R. Titova N. Mestre T. A. (2020). Therapy of Parkinson's disease subtypes. Neurotherapeutics 17, 1366–1377. 10.1007/s13311-020-00894-7 32749651 PMC7851253

[B68] Martın-Ruiz R. Puig M. V. Celada P. Shapiro D. A. Roth B. L. Mengod G.. (2001). Control of serotonergic function in medial prefrontal cortex by serotonin-2a receptors through a glutamate-dependent mechanism. J. Neurosci. 21, 9856–9866. 10.1523/JNEUROSCI.21-24-09856.2001 11739593 PMC6763049

[B69] Miguelez C. Grandoso L. Ugedo L. (2011). Locus coeruleus and dorsal raphe neuron activity and response to acute antidepressant administration in a rat model of Parkinson's disease. Int. J. Neuropsychopharmacol. 14, 187–200. 10.1017/S146114571000043X 20426885

[B70] Miguelez C. Navailles S. Deurwaerdère P. D. Ugedo L. (2016). The acute and long-term l-dopa effects are independent from changes in the activity of dorsal raphe serotonergic neurons in 6-ohda lesioned rats. Br. J. Pharmacol. 173, 2135–2146. 10.1111/bph.13447 26805402 PMC4908202

[B71] Miyanishi H. Suga S. Sumi K. Takakuwa M. Izuo N. Asano T.. (2023). The role of GABA in the dorsal striatum-raphe nucleus circuit regulating stress vulnerability in male mice with high levels of Shati/Nat8l. eNeuro 10, 1–14. 10.1523/ENEURO.0162-23.2023 37813564 PMC10598637

[B72] Nicholson S. L. Brotchie J. M. (2002). 5-hydroxytryptamine (5-ht, serotonin) and Parkinson's disease - opportunities for novel therapeutics to reduce the problems of levodopa therapy. Eur. J. Neurol. 9, 1–6. 10.1046/j.1468-1331.9.s3.1.x 12464115

[B73] Obeso J. A. Rodriguez-Oroz M. C. Goetz C. G. Marin C. Kordower J. H. Rodriguez M.. (2010). Missing pieces in the Parkinson's disease puzzle. Nat. Med. 16, 653–661. 10.1038/nm.2165 20495568

[B74] O'Callaghan C. Hezemans F. H. Ye R. Rua C. Jones P. S. Murley A. G.. (2021). Locus coeruleus integrity and the effect of atomoxetine on response inhibition in Parkinson's disease. Brain 144, 2513–2526. 10.1093/brain/awab142 33783470 PMC7611672

[B75] Oh Y.-S. Yoo S.-W. Lyoo C. H. Kim J.-S. (2022). Decreased thalamic monoamine availability in drug-induced parkinsonism. Sci. Rep. 12:3749. 10.1038/s41598-022-07773-5 35260679 PMC8904448

[B76] Pagonabarraga J. Kulisevsky J. Strafella A. P. Krack P. (2015). Apathy in Parkinson's disease: clinical features, neural substrates, diagnosis, and treatment. Lancet Neurol. 14, 518–531. 10.1016/S1474-4422(15)00019-8 25895932

[B77] Pardo-Moreno T. García-Morales V. Suleiman-Martos S. Rivas-Domínguez A. Mohamed-Mohamed H. Ramos-Rodríguez J. J.. (2023). Current treatments and new, tentative therapies for Parkinson's disease. Pharmaceutics 15:770. 10.3390/pharmaceutics15030770 36986631 PMC10051786

[B78] Pare D. Curro Dossi R. Steriade M. (1990). Neuronal basis of the parkinsonian resting tremor: a hypothesis and its implications for treatment. Neuroscience 35, 217–226. 10.1016/0306-4522(90)90077-H 2199839

[B79] Parker J. G. Marshall J. D. Ahanonu B. Wu Y.-W. Kim T. H. Grewe B. F.. (2018). Diametric neural ensemble dynamics in parkinsonian and dyskinetic states. Nature 557, 177–182. 10.1038/s41586-018-0090-6 29720658 PMC6526726

[B80] Pasquini J. Ceravolo R. Qamhawi Z. Lee J.-Y. Deuschl G. Brooks D. J.. (2018). Progression of tremor in early stages of Parkinson's disease: a clinical and neuroimaging study. Brain 141, 811–821. 10.1093/brain/awx376 29365117

[B81] Perez-Lloret S. Barrantes F. J. (2016). Deficits in cholinergic neurotransmission and their clinical correlates in Parkinson's disease. NPJ Parkinsons Dis. 2:16001. 10.1038/npjparkd.2016.1 28725692 PMC5516588

[B82] Peyron C. Luppi P.-H. Fort P. Rampon C. Jouvet M. (1996). Lower brainstem catecholamine afferents to the rat dorsal raphe nucleus. J. Comp. Neurol. 364, 402–413. 10.1002/(SICI)1096-9861(19960115)364:3&lt;402::AID-CNE2&gt;3.0.CO;2-8 8820873

[B83] Pirker W. Katzenschlager R. Hallett M. Poewe W. (2023). Pharmacological treatment of tremor in Parkinson's disease revisited. J. Parkinsons Dis. 13:127. 10.3233/JPD-225060 36847017 PMC10041452

[B84] Politis M. Niccolini F. (2015). Serotonin in Parkinson's disease. Behav. Brain Res. 277, 136–145. 10.1016/j.bbr.2014.07.037 25086269

[B85] Prange S. Klinger H. Laurencin C. Danaila T. Thobois S. (2022). Depression in patients with Parkinson's disease: current understanding of its neurobiology and implications for treatment. Drugs Aging 39, 417–439. 10.1007/s40266-022-00942-1 35705848 PMC9200562

[B86] Press W. H. (Ed.). (2007). Numerical Recipes: The Art of Scientific Computing, 3rd Edn. Cambridge: Cambridge University Press.

[B87] Prinz A. Selesnew L.-M. Liss B. Roeper J. Carlsson T. (2013). Increased excitability in serotonin neurons in the dorsal raphe nucleus in the 6-ohda mouse model of Parkinson's disease. Exp. Neurol. 248, 236–245. 10.1016/j.expneurol.2013.06.015 23810738

[B88] Ranjbar-Slamloo Y. Fazlali Z. (2020). Dopamine and noradrenaline in the brain; overlapping or dissociate functions? Front. Mol. Neurosci. 12:334. 10.3389/fnmol.2019.00334 32038164 PMC6986277

[B89] Rav-Acha M. Bergman H. Yarom Y. (2008). Pre- and postsynaptic serotoninergic excitation of globus pallidus neurons. J. Neurophysiol. 100, 1053–1066. 10.1152/jn.00845.2007 18550726

[B90] Reed M. C. Nijhout H. F. Best J. (2013). Computational studies of the role of serotonin in the basal ganglia. Front. Integr. Neurosci. 7:41. 10.3389/fnint.2013.00041 23745108 PMC3663133

[B91] Riley K. F. Hobson M. P. Bence S. J. (2006). Mathematical Methods for Physics and Engineering, 3rd Edn. Cambridge: Cambridge University Press. 10.1017/CBO9780511810763

[B92] Rommelfanger K. Weinshenker D. (2007). Norepinephrine: the redheaded stepchild of Parkinson's disease. Biochem. Pharmacol. 74, 177–190. 10.1016/j.bcp.2007.01.036 17416354

[B93] Segal M. (1979). Serotonergic innervation of the locus coeruleus from the dorsal raphe and its action on responses to noxious stimuli. J. Physiol. 286, 401–415. 10.1113/jphysiol.1979.sp012628 439032 PMC1281580

[B94] Severson K. A. Chahine L. M. Smolensky L. A. Dhuliawala M. Frasier M. Ng K.. (2021). Discovery of Parkinson's disease states and disease progression modelling: a longitudinal data study using machine learning. Lancet Digit. Health 3, e555–e564. 10.1016/S2589-7500(21)00101-1 34334334

[B95] Shi Z. Yao W. Li Z. Zeng L. Zhao Y. Zhang R.. (2020). Artificial intelligence techniques for stability analysis and control in smart grids: methodologies, applications, challenges and future directions. Appl. Energy 278:115733. 10.1016/j.apenergy.2020.115733

[B96] Szabo S. T. Blier P. (2001). Functional and pharmacological characterization of the modulatory role of serotonin on the firing activity of locus coeruleus norepinephrine neurons. Brain Res. 922, 9–20. 10.1016/S0006-8993(01)03121-3 11730697

[B97] Tan Y.-Y. Jenner P. Chen S.-D. (2022). Monoamine oxidase-b inhibitors for the treatment of Parkinson's disease: past, present, and future. J. Parkinsons Dis. 12, 477–493. 10.3233/JPD-212976 34957948 PMC8925102

[B98] Temel Y. Boothman L. J. Blokland A. Magill P. J. Steinbusch H. W. M. Visser-Vandewalle V.. (2007). Inhibition of 5-ht neuron activity and induction of depressive-like behavior by high-frequency stimulation of the subthalamic nucleus. Proc. Nat. Acad. Sci. 104, 17087–17092. 10.1073/pnas.0704144104 17942692 PMC2040465

[B99] Tozzi A. Sciaccaluga M. Loffredo V. Megaro A. Ledonne A. Cardinale A.. (2021). Dopamine-dependent early synaptic and motor dysfunctions induced by α-synuclein in the nigrostriatal circuit. Brain 144, 3477–3491. 10.1093/brain/awab242 34297092 PMC8677552

[B100] Vermeiren Y. Deyn P. P. D. (2017). Targeting the norepinephrinergic system in Parkinson's disease and related disorders: the locus coeruleus story. Neurochem. Int. 102, 22–32. 10.1016/j.neuint.2016.11.009 27899296

[B101] Vertes R. P. (1991). A pha-l analysis of ascending projections of the dorsal raphe nucleus in the rat. J. Comp. Neurol. 313, 643–668. 10.1002/cne.903130409 1783685

[B102] Voon V. Fox S. H. (2007). Medication-related impulse control and repetitive behaviors in Parkinson's disease. Arch. Neurol. 64:1089. 10.1001/archneur.64.8.1089 17698698

[B103] Wang S. Zhang Q. Liu J. Wu Z. Wang T. Gui Z.. (2009). Unilateral lesion of the nigrostriatal pathway induces an increase of neuronal firing of the midbrain raphe nuclei 5-HT neurons and a decrease of their response to 5-HT1A receptor stimulation in the rat. Neuroscience 159, 850–861. 10.1016/j.neuroscience.2008.12.051 19174182

[B104] Williams-Gray C. H. Foltynie T. Brayne C. E. G. Robbins T. W. Barker R. A. (2007). Evolution of cognitive dysfunction in an incident Parkinson's disease cohort. Brain 130, 1787–1798. 10.1093/brain/awm111 17535834

[B105] Wilson H. Dervenoulas G. Pagano G. Koros C. Yousaf T. Picillo M.. (2019). Serotonergic pathology and disease burden in the premotor and motor phase of A53T α-synuclein parkinsonism: a cross-sectional study. Lancet Neurol. 18, 748–759. 10.1016/S1474-4422(19)30140-1 31229470

[B106] Wu T. Hallett M. (2013). The cerebellum in Parkinson's disease. Brain 136, 696–709. 10.1093/brain/aws360 23404337 PMC7273201

[B107] Ye R. Hezemans F. H. O'Callaghan C. Tsvetanov K. A. Rua C. Jones P. S.. (2023). Locus coeruleus integrity is linked to response inhibition deficits in Parkinson's disease and progressive supranuclear palsy. J. Neurosci. 43, 7028–7040. 10.1523/JNEUROSCI.0289-22.2023 37669861 PMC10586538

[B108] Zach H. Dirkx M. F. Roth D. Pasman J. W. Bloem B. R. Helmich R. C.. (2020). Dopamine-responsive and dopamine-resistant resting tremor in Parkinson's disease. Neurology 95, e1461–e1470. 10.1212/WNL.0000000000010316 32651292

[B109] Zhang H. Li K. Chen H.-S. Gao S.-Q. Xia Z.-X. Zhang J.-T.. (2018). Dorsal raphe projection inhibits the excitatory inputs on lateral habenula and alleviates depressive behaviors in rats. Brain Struct. Funct. 223, 2243–2258. 10.1007/s00429-018-1623-3 29460052

[B110] Zhang X. Cui N. Wu Z. Su J. Tadepalli J. S. Sekizar S.. (2010). Intrinsic membrane properties of locus coeruleus neurons in *mecp2*-null mice. Am. J. Physiol.-Cell Physiol. 298, C635–C646. 10.1152/ajpcell.00442.2009 20042730 PMC2838567

