# Supplementary material for: Simulating combined monoaminergic depletions in a PD animal model through a bio-constrained differential equations system
Source: Front Comput Neurosci. 2024 Aug 23;18:1386841. doi: 10.3389/fncom.2024.1386841 (PMC11378529; doi:10.3389/fncom.2024.1386841)
Supplement: Supplementary file 1 [file Data_Sheet_1.pdf]

Simulating combined monoaminergic depletions in a PD animal model through a bio-constrained differential equations system

Samuele Carli [1,2,3,4], **Luigi Brugnano** [3] **and Daniele Caligiore** [1,4]

[1] *Computational and Translational Neuroscience Laboratory, Institute of Cognitive Sciences and Technologies, National Research Council (CTNLab-ISTC-CNR), Via Giandomenico Romagnosi 18a, Rome 00196 Italy*
[2] *Entersys s.r.l., Via San Pio X 44 Noventa Padovana (Padua), 35027 Italy*
[3] *Department of Mathematics and Computer Science "U. Dini", University of Florence, Viale Morgagni 67/A, Florence, 50134 Italy*
[4] *AI2Life s.r.l., Innovative Start-Up, ISTC-CNR Spin-Off, Via Sebino 32, Rome 00199 Italy*

Correspondence*:
Daniele Caligiore
daniele.caligiore@istc.cnr.it

1 APPENDIX 1: STABILITY CONDITIONS

Let us consider a nonlinear system $\dot{\mathbf{y}} = f(t, \mathbf{y})$, with initial conditions $\mathbf{y}(t_0) = \mathbf{y}_0$ and which is assumed to admit an equilibrium point in $\bar{\mathbf{y}} = \mathbf{0}$[1] The null equilibrium point is *stable* if:

$$\forall \epsilon > 0 \; \exists \delta = \delta(\epsilon, t_0) \text{ such that } \|\mathbf{y}_0\| \leq \delta \Rightarrow \|\mathbf{y}(t)\| \leq \epsilon, \quad \forall t \geq t_0 \tag{1}$$

or in words, a neighborhood of the equilibrium point exists such that any initial point from that neighbourhood remains arbitrarily close to the equilibrium point.

More stringently, we can ask for the neighborhood to be constant with respect to time: if $\delta = \delta(\epsilon)$, the solution is *uniformly stable*.

If also $\lim_{t\to\infty} \mathbf{y}(t) = \mathbf{0}$ holds, the solution is *asymptotically stable*.

An even more stringent requirement defines an *exponentially asymptotically stable* solution:

$$\exists \alpha \geq 1, \; \beta, \delta > 0, \text{ such that } \|\mathbf{y}_0\| \leq \delta \Rightarrow \|\mathbf{y}(t)\| \leq \alpha\delta e^{-\beta(t-t_0)}, \; \forall t \geq t_0. \tag{2}$$

System (9):

$$\dot{\mathbf{y}}(t) = A\mathbf{y}(t) + C(\mathbf{y}(t) \circ \mathbf{y}(t)) + \mathbf{b} = f(t, \mathbf{y}) \tag{3}$$

can be translated to have an equilibrium point at the origin. Let $\bar{\mathbf{y}}$ be the equilibrium point, such that:

$$\mathbf{0} = A\bar{\mathbf{y}} + C(\bar{\mathbf{y}} \circ \bar{\mathbf{y}}) + \mathbf{b} = f(t, \bar{\mathbf{y}}) \tag{4}$$

[1] a point such that $\dot{\mathbf{y}} = f(t, \mathbf{0}) = \mathbf{0}$

We obtain:

$$\begin{aligned}(\mathbf{y}+\bar{\mathbf{y}})' = \dot{\mathbf{y}} &= A(\mathbf{y}+\bar{\mathbf{y}}) + C((\mathbf{y}+\bar{\mathbf{y}})\circ(\mathbf{y}+\bar{\mathbf{y}})) + \mathbf{b} \\ &= A\mathbf{y} + A\bar{\mathbf{y}} + C(\mathbf{y}\circ\mathbf{y}) + 2C(\mathbf{y}\circ\bar{\mathbf{y}}) + C(\bar{\mathbf{y}}\circ\bar{\mathbf{y}}) + \mathbf{b} \\ &= A\mathbf{y} + C(\mathbf{y}\circ\mathbf{y}) + 2C(\mathbf{y}\circ\bar{\mathbf{y}}) \end{aligned} \tag{5}$$

since (4) holds. Now $\mathbf{y} = \mathbf{0}$ is clearly an equilibrium point, since A and C are linear combinations and an element-wise product by the zero vector is zero.

Let $D_{\bar{\mathbf{y}}} = \mathrm{diag}(\bar{\mathbf{y}})$; we can now rewrite $\mathbf{y}\circ\bar{\mathbf{y}}$ as $D_{\bar{\mathbf{y}}}\mathbf{y}$. We therefore have:

$$\dot{\mathbf{y}} = A\mathbf{y} + C(\mathbf{y}\circ\mathbf{y}) + 2CD_{\bar{\mathbf{y}}}\mathbf{y} = (A + 2CD_{\bar{\mathbf{y}}})\mathbf{y} + C(\mathbf{y}\circ\mathbf{y}) \tag{6}$$

which can be represented as a sum of a linear and a nonlinear term by setting $\tilde{A} = (A + 2CD_{\bar{\mathbf{y}}})$ and $g(\mathbf{y}) = C(\bar{\mathbf{y}}\circ\bar{\mathbf{y}})$:

$$\dot{\mathbf{y}}(t) = \tilde{A}\mathbf{y}(t) + \mathbf{g}(\mathbf{y}(t)) \tag{7}$$

since matrices $A, C, D_{\bar{\mathbf{y}}}$ are constants with respect to time in (6).

It is now possible to check the applicability of Perron's theorem ((Brugnano, 2018, p.132)), which states that given a system in the form $\dot{\mathbf{y}}(t) = A\mathbf{y}(t) + \mathbf{g}(t, \mathbf{y}(t))$, if $\sigma(A) \subset \mathbb{C}^-$ and:

$$\lim_{\|\mathbf{y}\|\to 0} \frac{\|\mathbf{g}(t,\mathbf{y})\|}{\|\mathbf{y}\|} = 0 \tag{8}$$

uniformly with respect to t, then $\mathbf{y} = \mathbf{0}$ is exponentially asymptotically stable.

Since:

$$\|\mathbf{g}(\mathbf{y})\| = \|C(\mathbf{y}\circ\mathbf{y})\| \tag{9}$$

condition (8) is clearly satisfied, hence if also $\sigma(\tilde{A}) \subset \mathbb{C}^-$ holds, the system is exponentially asymptotically stable.

Assuming A, C and $\mathbf{b}$ to be known for a particular instance of system (9), it is therefore necessary to compute an approximation of the equilibrium point $\bar{\mathbf{y}}$ and, consequently, $\tilde{A}$:

$$\tilde{A} = A + 2CD_{\bar{\mathbf{y}}}, \quad D_{\bar{\mathbf{y}}} = \mathrm{diag}(\bar{\mathbf{y}}) \tag{10}$$

before the stability condition can be verified.

In the particular case of system (1)-(6), C has only one nonzero element, and therefore:

$$2CD_{\bar{\mathbf{y}}} = 2(c_{46}\mathbf{e}_4)(\bar{y}_6\mathbf{e}_6^T) = 2\beta^{\mathrm{LC}}_{\mathrm{SNcVTA}}\bar{y}_6\mathbf{e}_4\mathbf{e}_6^T \tag{11}$$

where $\mathbf{e}_k$ is as usual the k-th versor of the canonical base.

The equilibrium point $\bar{\mathbf{y}}$ can be approximated by applying the Newton method to find the root of:

$$f(\mathbf{y}) = A\mathbf{y} + C(\mathbf{y}\circ\mathbf{y}) + \mathbf{b} \tag{12}$$

using the iteration:

$$\begin{aligned}\bar{\mathbf{y}}^{l+1} &= \bar{\mathbf{y}}^l - f'(\bar{\mathbf{y}}^l)^{-1} f(\bar{\mathbf{y}}^l) \\ &= \bar{\mathbf{y}}^l - (A + 2CD_{\bar{\mathbf{y}}^l})^{-1} f(\bar{\mathbf{y}}^l)\end{aligned} \tag{13}$$

and taking as the starting point $\bar{\mathbf{y}}^0$ the solution of $A\bar{\mathbf{y}} + \mathbf{b} = \mathbf{0}$. The system is therefore exponentially asymptotically stable if both $\sigma(A) \subset \mathbb{C}^-$ and $\sigma(\tilde{A}) \subset \mathbb{C}^-$ are verified.
The iteration to compute the equilibrium point can be stopped when:

$$\max_i |\bar{y}_i^{l+1} - \bar{y}_i^l| < \text{tol} \tag{14}$$

hence when the maximum error on each component of $\bar{\mathbf{y}}^{l+1}$ is smaller than a prescribed tolerance.

2 APPENDIX 2: DETAILS OF THE MODEL FITNESS MEASURES

We consider a fitness measure to be a single figure of merit normalized to $[0, 1]$, that summarises how close the model is to achieving a set of aims. In this section we identify a fitness measure suitable for system (9) and the corresponding lesioned variations described in Section (2.3).

2.1 Fitness measure

The fitness measure for subject S_i is a composition of many fitness. To completely describe the model of a subject, other than its set of parameters S_i, we need to represent its corresponding set of target values T_i: T_i contains the reference solutions that the model is supposed to reproduce when using the parameters in S_i; T_i must therefore have one reference solution for each of the states (healthy and lesions) that we are modelling.
In particular, we assume that we can address a particular reference solution from T_i in a similar way to the solution corresponding to particular subsets of parameters in S_i:

$$T_i^{SHAM}(J) = Y_T(J) = \begin{pmatrix} y_{T1}(t_0) & \cdots & y_{T1}(t_N) \\ \vdots & & \vdots \\ y_{Ts}(t_0) & \cdots & y_{Ts}(t_N) \end{pmatrix} \tag{15}$$

under the assumption that T_i can provide the reference solution for any discrete set of times $J = \{t_0, ..., t_N\}$ which is decided by the integration algorithm during the computation of the solution $S_i^{\text{SHAM}}(\mathbf{y}_0, t_0, T)$. The same notation of course applies for the other cases, T_i^{LDA}, T_i^{L5HT} and so on.

The subject index will intentionally be left out in the following sections to lighten the notation further, since it's not relevant in the context: the fitness is of course computed independently for each subject in the same way.

2.2 SHAM fitness

The fitness of the healthy instance is divided in one fitness measure for each equation.
To simplify the notation, we define:

- $Y_T = T^{SHAM}(J)$, the corresponding target solution
- $\mathbf{y}_0 = T^{SHAM}(t_0)$ the starting vector
- $Y = S^{\text{SHAM}}(\mathbf{y}_0, t_0, T)$, the simulation of the model using the appropriate subset of parameters
- $J = \{t_0, ..., t_N \leq T\}$, the time base chosen by the integration method

For each of the s equations in the status vector we can compute the corresponding mse_i:

$$\text{mse}_i = \sum_{j=1}^{N} (t_j - t_{j-1}) e_{ij}^2, \quad (e)_{ij} = Y_T - Y, \quad i = 1, .., s \tag{16}$$

and finally the simulation-time-weighted fitness:

$$f_i^{SHAM} = \frac{t_N - t_0}{T - t_0} \frac{1}{1 + mse_i} \tag{17}$$

The set of measures for the SHAM instance is therefore:

$$F^{SHAM} = \{f_i^{SHAM} | i = 1, ..., s\} \tag{18}$$

2.3 LDA fitness

Similarly to the SHAM case, we define:

- $Y_T = T^{LDA}(J)$, the corresponding target solution
- $\mathbf{y}_{h0} = T^{SHAM}(t_0)$ the starting vector for the healthy case
- $\mathbf{y}_{l0} = T^{LDA}(t_0)$ the starting vector for the LDA case
- $Y = S^{\text{LDA}}(\mathbf{y}_0, t_0, T)$, the simulation of the model using the appropriate subset of parameters
- $J = \{t_0, ..., t_N \leq T\}$, the time base chosen by the integration method
- $t_c = \frac{T - t_0}{2}$, the time before which we ignore the solution's fitness

As motivated in Section (3.3), in this case we have only three reference solutions to consider: GP,SNcVTA and LC; in particular, the former is to be fitted exactly from experimental data. The SNcVTA fitness is a conceptual requirement since we don't have the corresponding exact experimental data: LDA is a lesion of neurons in SNcVTA that in turn lowers the levels of dopamine. We therefore require that the average activation frequency of SNcVTA has to become at least as low as indicated in the reference solution, but can be free to become even lower. Similarly, available data suggests a lowered activity in LC to be at most 80% of the healthy value.
We also require the solution to be stable with two different initial conditions: the solution should obviously be stable near the equilibrium point (the LDA solution $\mathbf{y}_{l0}$), but perhaps more importantly, a healthy subject (hence starting with $\mathbf{y}_{h0}$) must be able to transition to the lesioned state (as it naturally occurs in-vivo during the experiment) without incurring in instabilities. This also implies that the first transient phase should be ignored in the computation of the fitness; for that reason we defined t_c as a threshold time before which we ignore the solution. Particular care should be used in choosing t_0, T and consequently t_c big enough with respect to the time constants of the system.
We therefore define two fitness measures for GP:

$$f_{\text{GP}}^{LDA}(\mathbf{y}_{l0}, t0, T, t_c), \quad f_{\text{GP}}^{LDA}(\mathbf{y}_{h0}, t0, T, t_c) \tag{19}$$

both are computed as in (18):

$$f_{\text{GP}}^{LDA}(\mathbf{y}_{l0}, t0, T, t_c), \quad f_{\text{GP}}^{LDA}(\mathbf{y}_{h0}, t0, T, t_c) \tag{20}$$

$$f_i^{LDA}(\mathbf{y}, t_0, T, t_c) = \frac{t_N - t_0}{T - t_0} \frac{1}{1 + mse_i}, \tag{21}$$

$$\text{mse}_i = \sum_{j=c}^{N} (t_j - t_{j-1}) e_{ij}^2, \quad (e)_{ij} = Y_T - Y, \tag{22}$$

where Y, Y_T and hence J are of course computed accordingly to the selected $\mathbf{y}$, c is the index of the first $t \geq t_c$ in J, and the index of GP in the status vector happens to be 1, hence $i = 1$.

The fitness $f^{LDA}_{\mathrm{SNcVTA}}$ for SNcVTA is computed in a similar way, but also applying a sieve function. that ignores negative errors:

$$f_i^{LDA}(\mathbf{y}, t_0, T, t_c) = \frac{t_N - t_0}{T - t_0}\frac{1}{1 + mse_i}, \tag{23}$$

$$\mathrm{mse}_i = \sum_{j=c}^{N}(t_j - t_{j-1})\max(0, e_{ij})^2, \quad (e)_{ij} = Y_T - Y, \tag{24}$$

where i is the index of the SNcVTA equation in the status vector.

Following the same principles, we define the two fitness measures for LC:

$$f_i^{LDA}(\mathbf{y}, t_0, T, t_c) = \frac{t_N - t_0}{T - t_0}\frac{1}{1 + mse_i}, \tag{25}$$

$$\mathrm{mse}_i = \sum_{j=c}^{N}(t_j - t_{j-1})\max(0, e_{ij})^2, \quad (e)_{ij} = Y_T - Y, \tag{26}$$

where this time i is the index of the LC equation in the status vector.

The set of measures for the LDA case therefore has four elements:

$$\begin{aligned} F^{LDA} = \{&f^{LDA}_{\mathrm{GP}}(\mathbf{y}_{l0}, t0, T, t_c), f^{LDA}_{\mathrm{GP}}(\mathbf{y}_{h0}, t0, T, t_c), \\ &f^{LDA}_{\mathrm{SNcVTA}}(\mathbf{y}_{l0}, t0, T, t_c), f^{LDA}_{\mathrm{SNcVTA}}(\mathbf{y}_{h0}, t0, T, t_c), \\ &f^{LDA}_{\mathrm{LC}}(\mathbf{y}_{l0}, t0, T, t_c), f^{LDA}_{\mathrm{LC}}(\mathbf{y}_{h0}, t0, T, t_c)\} \end{aligned} \tag{27}$$

2.4 L5HT and LNE fitness

The fitness measures for this two instances are conceptually identical to the LDA case; the fitness for GP is therefore computed according to (21), and the fitness for the lesioned areas use the the same sieve as in (23) (but of course selecting the correct lesioned area, respectively DRN and LC). The sets of measures for this two instances are defined as:

$$\begin{aligned} F^{L5HT} = \{&f^{L5HT}_{\mathrm{GP}}(\mathbf{y}_{l0}, t0, T, t_c), f^{L5HT}_{\mathrm{GP}}(\mathbf{y}_{h0}, t0, T, t_c), \\ &f^{L5HT}_{\mathrm{DRN}}(\mathbf{y}_{l0}, t0, T, t_c), f^{L5HT}_{\mathrm{DRN}}(\mathbf{y}_{h0}, t0, T, t_c)\} \end{aligned} \tag{28}$$

$$\begin{aligned} F^{LNE} = \{&f^{LNE}_{\mathrm{GP}}(\mathbf{y}_{l0}, t0, T, t_c), f^{LNE}_{\mathrm{GP}}(\mathbf{y}_{h0}, t0, T, t_c), \\ &f^{LNE}_{\mathrm{LC}}(\mathbf{y}_{l0}, t0, T, t_c), f^{LNE}_{\mathrm{LC}}(\mathbf{y}_{h0}, t0, T, t_c)\} \end{aligned} \tag{29}$$

2.5 Lesion combination fitness

The combination of lesions is left as unconstrained as possible to be able to serve as a prediction; we do however at least require the corresponding simulation not to diverge and to lie within an acceptable range. In the experiment lesions are applied in succession, LDA always first. It makes sense to require the solution to be stable with both $\mathbf{y}_{h0} = T^{SHAM}(t_0)$ and $\mathbf{y}_{l0} = T^{LDA}(t_0)$ as initial conditions. We define a fitness which only considers the temporal span of the solution to penalize early divergence:

$$f^{LDA+LNE}(\mathbf{y}, t_0, T) = \frac{t_N - t_0}{T - t_0}, \quad f^{LDA+L5HT}(\mathbf{y}, t_0, T) = \frac{t_N - t_0}{T - t_0} \tag{30}$$

where J and consequently t_N, t_0 are computed from the corresponding simulation $S^{LDA+lesion}(\mathbf{y}, t_0, T)$

Additionally, we require the GP value of LDA+L5HT to be within reasonable limits. In particular, we define specific limit fitness functions similar to (23), for example the lower bound:

$$f_{i,\min}^{LDA+L5HT}(\mathbf{y}, t_0, T, t_c) = \frac{t_N - t_0}{T - t_0} \frac{1}{1 + mse_i}, \tag{31}$$

$$\mathrm{mse}_i = \sum_{j=c}^{N} (t_j - t_{j-1}) \min(0, e_{ij})^2, \quad (e)_{ij} = Y_T - Y, \tag{32}$$

$$Y_T = T^{LDA+L5HT-\min}(J) \tag{33}$$

where i is again the index corresponding to GP and $T^{LDA+L5HT-\min}(J)$ is the reference solution containing the lower bound; likewise the upper bound will be defined similarly but using $\max$ as an error sieve against the upper bound reference solution $T^{LDA+L5HT-\max}(J)$. This limits are necessary to guide the optimization towards a solution which lies within the experimentally determined range and exclude instead solutions which may exhibit better fitness scores but are outside of the physiological range.

The set of measures for the combination of lesions is therefore:

$$\begin{aligned} F^{COMB} = \big\{ & f^{LDA+LNE}(\mathbf{y}_{h0}, t_0, T), f^{LDA+LNE}(\mathbf{y}_{l0}, t_0, T), \\ & f^{LDA+L5HT}(\mathbf{y}_{h0}, t_0, T), f^{LDA+L5HT}(\mathbf{y}_{l0}, t_0, T), \\ & f_{\mathrm{GP,min}}^{LDA+LNE}(\mathbf{y}_{h0}, t_0, T), f_{\mathrm{GP,min}}^{LDA+LNE}(\mathbf{y}_{l0}, t_0, T), \\ & f_{\mathrm{GP,max}}^{LDA+LNE}(\mathbf{y}_{h0}, t_0, T), f_{\mathrm{GP,min}}^{LDA+LNE}(\mathbf{y}_{l0}, t_0, T), \\ & f_{\mathrm{GP,max}}^{LDA+L5HT}(\mathbf{y}_{h0}, t_0, T), f_{\mathrm{GP,min}}^{LDA+L5HT}(\mathbf{y}_{l0}, t_0, T), \\ & f_{\mathrm{GP,max}}^{LDA+L5HT}(\mathbf{y}_{h0}, t_0, T), f_{\mathrm{GP,max}}^{LDA+L5HT}(\mathbf{y}_{l0}, t_0, T) \big\} \end{aligned} \tag{34}$$

2.6 Parameters constraints

The fitness function can also be useful to impose soft, dynamic constraints on the parameters. In this case, it makes sense to require the α^{ext} parameters of a lesion to be less or equal than its counterpart in the SHAM instance: that particular brain area have been damaged, and it makes sense to assume it would lower its average activation frequency in absence of other stimuli. We define the fitness measure:

$$f_l^{PAR} = \frac{1}{1 + max(0, S^l - S^{SHAM})} \tag{35}$$

where l is one of the three lesions (LDA, LNE, L5HT) and $S^l - S^{SHAM}$ represent the difference between the altered α^{ext} parameter in the lesioned subset and its counterpart in the healthy one.

As usual, we define the set:

$$F^{PAR} = \{ f_{LDA}^{PAR}, f_{LNE}^{PAR}, f_{L5HT}^{PAR} \} \tag{36}$$

2.7 Asymptotic stability constraints

Every subject state considered in this study is supposed to be stable in time; it is therefore important to impose that each set of parameters defines an asymptotically stable system which will ultimately never diverge from its equilibrium point. As shown in Section 2.4, system (1)-()6 is exponentially asymptotically stable if $\sigma(\tilde{A}) \subset \mathbb{C}^-$, where $A = A + 2CD_{\bar{\mathbf{y}}}$ (see (10)). We can therefore envisage a fitness measure:

$$f_l^{STAB} = \frac{1}{1 + \sum_i \max(0, \mathbb{R}(\lambda_i))} \tag{37}$$

where l is one of the parameter subsets which define the model (SHAM, LDA, etc.), and λ_i is an eigenvalue of the corresponding $\tilde{A}$. This measure will therefore always be 1 when the system is asymptotically stable, but tend to zero as the real part of the eigenvalues grows more positive.

The computation of $\tilde{A}$ requires using an iterative root-finding method to determine the equilibrium point $\bar{\mathbf{y}}$ of each parameters set $A, C, \mathbf{b}$. It is advantageous to use multiple stopping conditions for this method to avoid unnecessary computation, in particular:

- A tolerance on the precision of $\bar{\mathbf{y}}^l$ as defined in (14); this tolerance should be set to be compatible with the precision obtained with the parameters optimization algorithm. For example, if the optimization fitness required translates to an mse of 10^{-8}, it makes sense to require $\mathrm{tol} = 10^{-9}$.
- A arbitrary guard on the maximum number of allowed iterations; since precision is not of paramount importance in this context, the number of iterations can be kept rather small (≤ 25).
- A guard on the value of the components of $\bar{\mathbf{y}}$. If the method is converging to an equilibrium point which has some components which are too big or negative, the stability of the system is ultimately meaningless in the context of this study, therefore it is not worth investing computing power in obtaining it with high precision.

We finally define the set:

$$F^{STAB} = \{f^{STAB}_{SHAM}, f^{STAB}_{LDA}, f^{STAB}_{LNE}, f^{STAB}_{L5HT}, f^{STAB}_{LDA+LNE}, f^{STAB}_{LDA+L5HT}\} \quad (38)$$

2.8 The fitness, at last

We now have all the elements needed to compute the fitness of subject S_i.
Let F be the union of all the sets of measures we have defined:

$$F = F^{SHAM} \cup F^{LDA} \cup F^{L5HT} \cup F^{LNE} \cup F^{COMB} \cup F^{PAR} \cup F^{STAB} \quad (39)$$

we can now combine all the fitness measures we identified to obtain the total (or final) fitness figure:

$$f = \sqrt{\min_i(f_i)\frac{1}{n}\sum_{i=1}^{n} f_i}, \quad f_i \in F, \; n = |F| \quad (40)$$

REFERENCES

Brugnano, L. (2018). *Modelli numerici per la simulazione*
